# Olefin Metathesis in Confinement: Towards Covalent Organic Framework Scaffolds for Increased Macrocyclization Selectivity

**DOI:** 10.1002/chem.202104108

**Published:** 2022-01-05

**Authors:** Sebastian T. Emmerling, Felix Ziegler, Felix R. Fischer, Roland Schoch, Matthias Bauer, Bernd Plietker, Michael R. Buchmeiser, Bettina V. Lotsch

**Affiliations:** ^1^ Nanochemistry Department Max Planck Institute for Solid State Research Heisenbergstraße 1 70569 Stuttgart Germany; ^2^ Department of Chemistry University of Munich (LMU) Butenandtstraße 5–13 81377 Munich Germany; ^3^ Institute of Polymer Chemistry University of Stuttgart Pfaffenwaldring 55 70569 Stuttgart Germany; ^4^ Faculty of Chemistry and Food Chemistry Technical University of Dresden Bergstrasse 66 01069 Dresden Germany; ^5^ Department of Chemistry Faculty of Science and Center for Sustainable Systems Design (CSSD) Paderborn University Warburger Str. 100 33098 Paderborn Germany; ^6^ E-conversion Lichtenbergstraße 4a 85748 Garching Germany

**Keywords:** catalysis, confinement, covalent organic frameworks, metathesis, reticular chemistry

## Abstract

Covalent organic frameworks (COFs) offer vast structural and chemical diversity enabling a wide and growing range of applications. While COFs are well‐established as heterogeneous catalysts, so far, their high and ordered porosity has scarcely been utilized to its full potential when it comes to spatially confined reactions in COF pores to alter the outcome of reactions. Here, we present a highly porous and crystalline, large‐pore COF as catalytic support in α,ω‐diene ring‐closing metathesis reactions, leading to increased macrocyclization selectivity. COF pore‐wall modification by immobilization of a Grubbs‐Hoveyda‐type catalyst via a mild silylation reaction provides a molecularly precise heterogeneous olefin metathesis catalyst. An increased macro(mono)cyclization (MMC) selectivity over oligomerization (O) for the heterogeneous COF‐catalyst (MMC:O=1.35) of up to 51 % compared to the homogeneous catalyst (MMC:O=0.90) was observed along with a substrate‐size dependency in selectivity, pointing to diffusion limitations induced by the pore confinement.

## Graphical abstract

**A biomimetic** confinement and precise spatial arrangement of catalysts in porous media can provide increased selectivity for reactions compared to homogeneous catalysis. Herein, a covalent organic framework for molecular heterogeneous olefin metathesis is presented that utilizes spatial confinement of substrates in its pores for a substrate‐size dependent and increased macro(mono)cyclization selectivity.

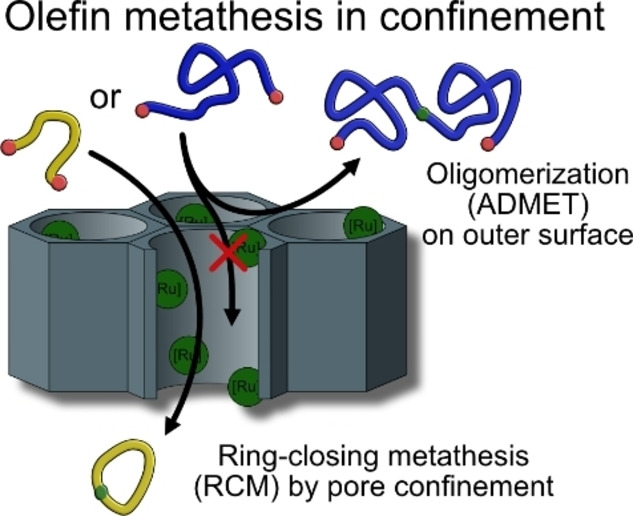

## Introduction

Covalent organic frameworks (COFs) are 2D or 3D extended structures, which are defined by their covalent connectivity, porosity, and crystallinity, while consisting exclusively of light elements.^[1]^ The vast structural and chemical diversity of COFs and the possibility to tune their framework with atomic precision has put COFs in the spotlight for a variety of applications that benefit from precise framework design, including photocatalytic water splitting,[^2^, ^3^] sensing,^[4]^ batteries,^[5]^ gas adsorption,^[6]^ or heterogeneous catalysis.^[7]^ With their ordered micro‐ and mesoporosity and large specific surface areas exposing a large number of functional or even active catalytic sites, COFs are among the most promising materials for molecular heterogeneous catalysis. Classical approaches to design COFs for heterogeneous catalysis utilizing this feature include incorporation of catalytic centers directly in the pore wall,^[8]^ pore surface engineering by molecular catalysts via a post‐synthetic reactions,^[9]^ integration of monodisperse nanoparticles in the framework by pore templating, or embedding polymers into the pores to combine multiple catalytic centers.[^10^, ^11^]

However, despite the promise of COFs as versatile scaffolds for catalysis, examples for the exploitation of reaction‐specific pore confinement effects during catalysis, such as the substrate‐ specific and size‐selective Knoevenagel‐reaction achieved for microporous COFs by Fang et al.,^[12]^ are still rare. Altering selectivity and reactivity of the catalyzed reaction by spatial confinement is an immensely successful principle used in nature by enzymes and enzyme‐inspired artificial catalysis. Taking full advantage of the ordered structural porosity of COFs thus bodes well for a biomimetic approach to catalysis where the precise spatial arrangement of catalytic centers and substrates, as well as pore confinement is utilized to direct product selectivity.^[13]^

**Scheme 1:**
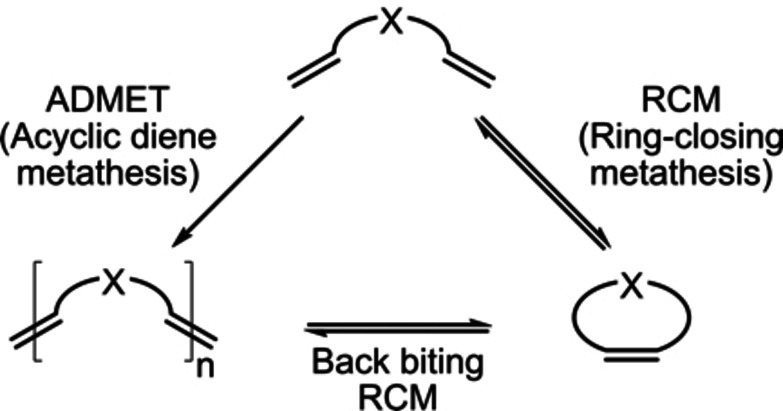
Competing metathesis reactions of α,ω‐dienes resulting in macro(mono)cycles and oligomerization products.

In this work, we present a large‐pore imine‐COF as a molecular heterogeneous catalyst to study the effect of spatial confinement on product selectivity during olefin metathesis reactions; in particular macro(mono)cyclization (MMC) selectivity by ring‐closing metathesis (RCM) and back‐biting depolymerization vs. acyclic diene metathesis (ADMET) oligomerization (O, Scheme 1).

Olefin metathesis‐based macrocyclization offers an important pathway to useful compounds for industrial or pharmaceutical chemistry,^[14]^ however, it still poses severe challenges. Oftentimes, only low MMC yields are achieved due to the competing oligomerization by ADMET, originating from a ring‐chain equilibrium during catalysis and back‐biting RCM.[^15^, ^16^] The biomimetic approach to olefin metathesis reactions by spatial confinement in pores for increased selectivity towards MMC products was already successfully shown for mesoporous silica by Jee et al. and Ziegler et al.[^16^, ^17^] Applying this biomimetic approach to a COFs system not only diversifies the scope of possible confinement effects and framework‐catalyst‐reactant interactions, but at the same time offers new opportunities for precise, substrate‐ and product‐specific catalyst‐framework designs due the high structural and chemical diversity of COFs.

## Results and Discussion

**Figure 1:**
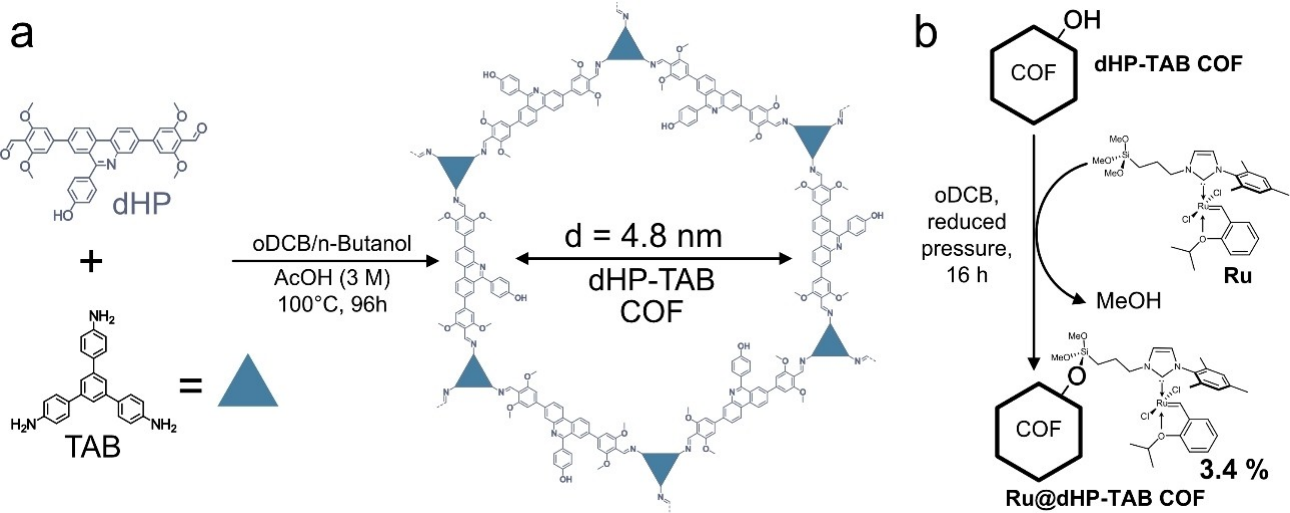
(a) Synthesis of **dHP**‐**TAB** COF. (b) Immobilization of **Ru** catalyst on **dHP‐TAB** by silylation to form **Ru@dHP**‐**TAB**.

For the study of olefin metathesis reactions under spatial confinement in COFs, the model system, **dHP**‐**TAB** COF, was synthesized by the condensation of 4,4′‐(6‐(4‐hydroxyphenyl)phenanthridine‐3,8‐diyl)bis(2,6‐dimethoxybenzal‐dehyde) (**dHP**) and 5′‐(4‐aminophenyl)‐[1,1′:3′,1′′‐terphenyl]‐4,4′′‐diamine (**TAB**) in a solvent mixture of 1,2‐dichlorobenzene (oDCB) and *n*‐butanol (3 : 7) with 3 M acetic acid (AcOH) as catalyst for 96 h at 100 °C (Figure 1, a). After isolation by filtration, the solid was washed with methanol and subsequently activated by supercritical CO_2_. The large‐pore COF system was chosen to accommodate the bulky catalyst and substrates and prevent pore blocking during immobilization and catalysis. Methoxy groups incorporated in the COF act as non‐covalent anchors to achieve better layer registry and thus high porosity and large, well‐defined pore sizes for this framework.^[18]^

Imine formation during the initial COF synthesis was confirmed by FTIR analysis (Figure S7). The spectrum shows the absence of the prominent aldehyde C=O stretching band at 1674 cm^−1^ and amine N−H stretching bands at 3355 cm^−1^ and 3431 cm^−1^, corresponding to the starting materials, indicating full conversion into imine bonds. The new imine stretching band is mostly concealed as slight shoulder at around 1614 cm^−1^ of the strong aromatic C−C stretching bands at 1593 cm^−1^. The solid‐state ^13^C nuclear magnetic resonance spectrum (CP‐MAS ssNMR) confirms the successful condensation by showing the typical imine signal at 160.0 ppm and the absence of aldehyde signals (Figure S18).

**Figure 2:**
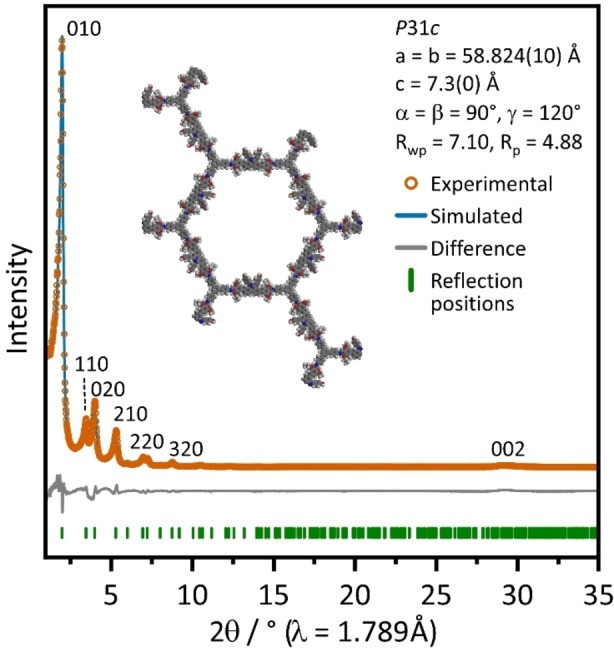
Experimental XRPD pattern of **dHP**‐**TAB** COF, Rietveld refinement,^[19]^ difference curve and positions of the Bragg reflections. Inset: Structure of the respective $A\bar{A}$ stacked **dHP**‐**TAB** along the *a* and *b* axis after refinement.

Crystallinity of **dHP‐TAB** COF was confirmed by X‐ray powder diffraction (XRPD), with the pattern (Co‐K_α1_) displaying several well‐resolved diffraction peaks at 2 *θ*=2.0°, 3.5°, 4.0°, 5.3°, 6.9°, 7.3°, 8.8°, 10.5° and a broadened stacking reflection centered around 28.5° (Figure 2).

The structure was modeled in *P*31*c* symmetry with an alternating nearly‐eclipsed $A\bar{A}$ stacking order of the layers. This assumed model is based on previous findings for phenylphenanthridine based COFs and shows a good match when compared to the simulated patterns of an $A\bar{A}$ stacked model (Figure S10). ^[18]^ Rietveld refinement of the pattern with the assumed model yielded unit cell parameters (*a*=*b*=58.824 Å and *c*=7.3 Å) with a satisfying agreement factor (R_wp_=7.10 %, R_p_=4.88 %) (Figure 2).^[19]^

Porosity of **dHP‐TAB** COF was investigated by nitrogen physisorption measurements at 77 K, showing a type‐IV isotherm, which is typical for mesoporous systems. The Brunauer‐Emmett‐Teller surface area (S_BET_) was calculated to be 1702 m^2^ g^−1^ with a total pore volume of 2.12 cm^3^ g^−1^ at P/P_0_=0.95. The pore size distribution (PSD) was determined from the adsorption branch by quenched solid density functional theory (QSDFT) based on the carbon model for cylindrical pores. It shows a narrow PSD around 4.8 nm, which is in good agreement with the structure model and closely related, isoreticular COFs.^[18]^

Next, the molecular catalyst RuCl_2_(N‐mesityl‐N‐(3‐(trimethoxysilyl)prop‐1‐yl)‐imidazol‐2‐ylidene)(CH‐2‐(2‐PrO‐C_6_H_4_)) (**Ru**) was immobilized in the framework via silylation, utilizing the incorporated hydroxyl groups of the protruding phenols as anchor points (Figure 1, b). The immobilization was performed at room temperature in high‐boiling oDCB as solvent under reduced pressure. Performing the reaction under reduced pressure significantly increases the catalyst loading by removing the accruing methanol from the reaction mixture and driving the reaction towards the desired outcome, yielding the catalyst‐loaded **Ru@dHP**‐**TAB** COF. Inductively‐coupled plasma optical emission spectroscopy (ICP‐OES) of the washed and dried sample revealed a Ru‐content of c(Ru)=42.3 μmol/g for **Ru@dHP**‐**TAB** COF. This corresponds to a successful silylation of approximately 3.4 % of the hydroxyl groups contained in the COF or roughly one catalyst per pore at every tenth layer.

**Figure 3:**
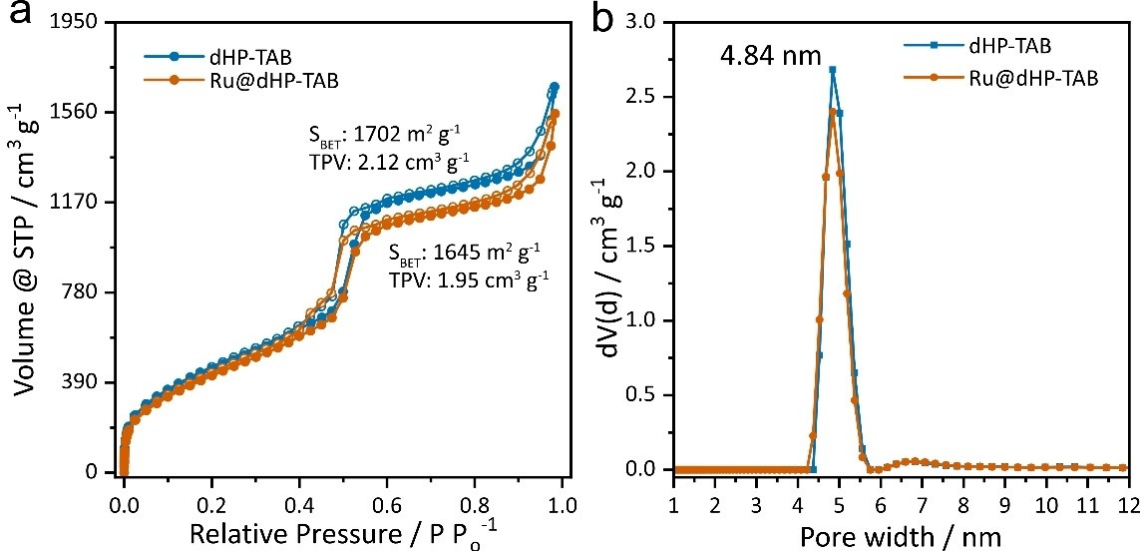
Comparison of (a) nitrogen isotherms at 77 K (filled circles for adsorption, empty circles for desorption) and (b) pore size distribution obtained from the adsorption branch of **dHP**‐**TAB** and **Ru@dHP**‐**TAB** after immobilization of the catalyst.

No changes in the FTIR spectra of **Ru@dHP**‐**TAB** compared to the pristine COF are visible, which can be attributed to the comparatively low amounts of immobilized catalyst (Figure S8) and the XRPD pattern (Figure S9); transmission electron microscopy (TEM) images show a retention of crystallinity (Figure S26). Nitrogen sorption measurements reveal only a minimal reduction in surface area (S_BET_=1645 m^2^ g^−1^) and total pore volume (1.95 cm^3^ g^−1^ at P/P_0_=0.95) compared to **dHP**‐**TAB** with nearly identical pore size distribution (Figure 3). The largely retained porosity suggests that no substantial fraction of pores was fully blocked during the immobilization.

To confirm the stability of the catalyst during immobilization and to gain knowledge of the catalyst‘s structure in the pore, X‐ray absorption (XAS) measurements were performed. Spectra of **Ru** and **Ru@dHP**‐**TAB** as solids and in solution/suspension (benzene), respectively, were recorded.

**Figure 4:**
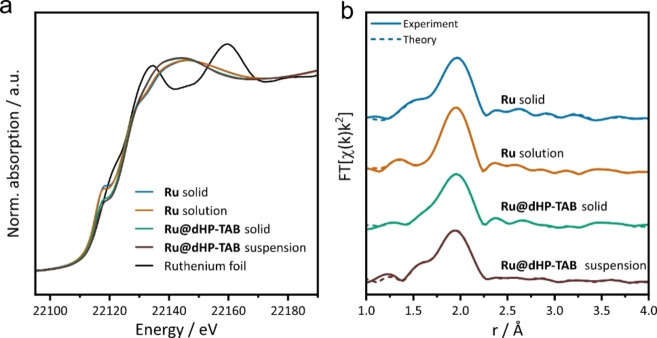
(a) XANES spectra of the homogeneous **Ru** complex in the solid state (red), solution (green), immobilized in the mesoporous COF in the solid state (yellow), as suspension in benzene (brown), as well as of the Ru(0) foil used for calibration (black). (b) Fourier‐transformed EXAFS data of the four Ru complexes. Continuous line: experimental data, dotted line: fitted data.

The obtained X‐ray absorption near edge structure (XANES) spectra (Figure 4, a and Figure S19), providing information about the oxidation state of the Ru‐metal center,^[20]^ show no differences in the edge energy for all four samples measured. A change in the electronic structure or oxidation state by immobilization in the COF can thus be excluded.

Results of extended X‐ray absorption fine structure (EXAFS) analysis, probing the local geometric structure around an X‐ray absorbing atom of the **Ru** catalyst under homogeneous and immobilized conditions are shown in Figure 4, b.^[21]^ The corresponding first shell scattering paths combined with coordination numbers, bond distances and Debye‐Waller factors, which describe the static and dynamic disorder in the coordination shell, are collected in Figure S21 and Table S7. The results of the structure analysis for all samples are in good agreement with the single‐crystal structure of the **Ru** catalyst.^[16]^ In the solid immobilized sample **Ru@dHP**‐**TAB**, the second Ru−C distance is slightly elongated compared to the pure **Ru** complex, and the coordination number of this shell is increased. Since the changes cannot be explained by major structural modifications, they are assigned to the effect of the immobilization, such as pore wall interactions. This conclusion is backed by the results for **Ru@dHP**‐**TAB** in benzene, where the structural alterations are reversed, as identified by structural parameters very similar to those of **Ru** in solution. Based on these observations, it can be concluded that neither the dissolution of the homogeneous complex in benzene nor the immobilization in a mesoporous COF lead to significant changes of the complex structure, which stays intact after immobilization. Neither ligand dissociation nor an association can be observed.

**Figure 5:**
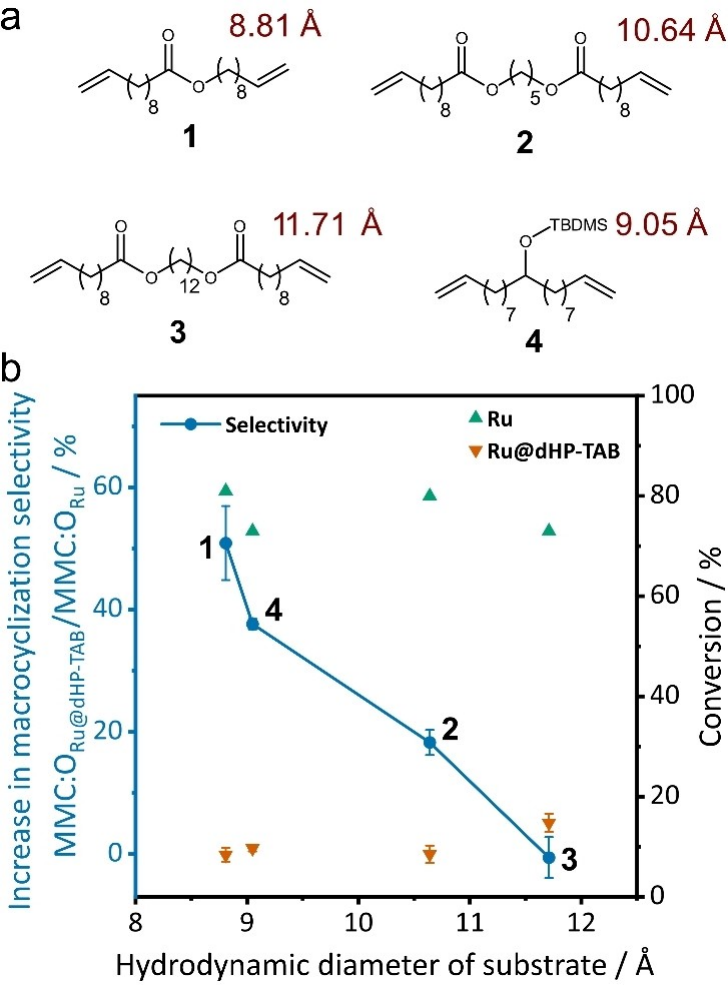
(a) Substrates **1**–**4** used in this study and their respective hydrodynamic radii (red) acc. to Ziegler *et al*.^[16]^ (b) Correlation between the hydrodynamic radii of the substrates and increase in macrocyclization selectivity (blue) and conversion rate for the homogeneous (green) and heterogeneous catalyst (orange). The average of three reactions with **Ru@dHP**‐**TAB** are displayed.

After confirmation of the stability of the COF framework and its immobilized catalyst, olefin metathesis reactions were carried out to determine the catalytic efficiency and the effect of the spatial confinement on MMC:O product selectivity. For this purpose, olefin metathesis reactions of four substrates (Figure 5, a) differing in their hydrodynamic radius and polarity were performed with the homogeneous **Ru** complex (Figure 1, b) as well as with **Ru@dHP**‐**TAB**. ^[16]^ The reactions were carried out under identical conditions at 50 °C for 16 h in C_6_D_6_ using 0.5‐mol % of catalyst and a substrate concentration of 25 mM; results are summarized in Table 1.

**Table 1:** Conversion, MMC:O ratio and selectivity for the RCM of substrate **1**–**4** by the action of **Ru** (0.5 mol‐%) and **Ru@dHP**‐**TAB** (0.5 mol‐%) at 50 °C as determined by NMR (Figure S2‐6).

Substrate	Conversion after 16 h [%]		MMC : O		MMC Selectivity [%]	
	Ru	Ru@ dHP‐TAB^[a]^	Ru	Ru@ dHP‐TAB^[a]^	Ru	Ru@ dHP‐TAB^[a]^
1	81	9	0.90	1.35	47	56
2	77	10	0.84	0.99	46	50
3	80	9	0.65	0.63	39	39
4	73	14	0.40	0.55	28	35

[a] Average over three performed reactions.

Stability of the COF framework during catalysis conditions was confirmed by post‐catalysis XRPD, nitrogen sorption, ICP‐OES and TEM measurements of the isolated materials, showing the retention of crystallinity and porosity (Figure S11, S15, S28). While the framework remains intact, the formation of unstable Ru methylidene complexes occurring during the catalysis ultimately deactivates the immobilized catalyst and therefore prevents recyclability.

The immobilization significantly alters the productivity of the catalyst, reducing the overall conversion after 16 h reaction time from around 80 % to around 10 %. This reduction was consistent for all substrates, independent of their size. This drastically reduced productivity is attributed to diffusion limitations and catalyst decomposition occurring during the reaction. However, a size‐dependent increase in selectivity MMC:O was found for the catalysis with **Ru@dHP**‐**TAB** compared to **Ru** (Figure 5, b). For the smallest substrate **1** (8.81 Å), an increase of 51 % in the MMC:O ratio from 0.90 for **Ru** to 1.35 for **Ru@dHP**‐**TAB** was found, corresponding to a 9 % increase in selectivity compared to the homogeneous catalyst system. Furthermore, a continuously reduced macro(mono)cyclization selectivity with increasing substrate size is observed. For substrate **4** (9.05 Å) with a very similar radius to **1** the MMC:O ratio is increased by 38 %.

The very similar increase in selectivity for **1** and **4** with almost identical size but different polarity suggests that the polarity of the substrate has little influence on the reaction outcome. For the second largest substrate **2** (10.64 Å) only 18 % are achieved and the largest substrate **3** (11.71 Å) shows the same selectivity when catalyzed by **Ru@dHP**‐**TAB** compared to the homogeneous **Ru**. This considerable size effect is rationalized by the substrate diffusion limitation into the COF mesoporous pores with increasing hydrodynamic radius. In the case of larger substrates, the reaction is mostly catalyzed by catalyst bound on the outer surface and close to the pore openings, mimicking the homogeneous ring‐chain equilibrium.^[15]^ Smaller substrates can diffuse more easily and penetrate into the COF pores more deeply, where pore confinement effects can take place, favoring RCM for the ring‐chain equilibrium products of the reaction by suppressing the formation of higher oligomers.[^22^, ^23^] This is likely due to the very large internal surface area of the highly porous COF material that offers enough “inner” pore surface area for this size selective confinement effect to take place and to alter the ring‐chain equilibrium.

## Conclusion

In summary, we have developed a phenylphenanthridine‐based COF containing accessible hydroxyl‐groups on its protruding phenyl groups that allowed the successful immobilization of a Hoveyda‐Grubbs‐type catalyst in its pores to study possible pore confinement effects on the MMC selectivity during olefin metathesis reactions. The structure and ordered porosity of large‐pore **dHP**‐**TAB** COF with a pore size of 4.8 nm, suitable to accommodate both the bulky molecular catalyst and nm‐sized substrates, was ascertained by XRPD analysis and nitrogen sorption experiments. The Ru‐catalyst was effectively immobilized by simple silylation on hydroxyl‐anchor groups integrated quantitatively in the framework and the retention of the catalyst's structure upon immobilization was confirmed by XANES/EXAFS measurements. A set of four diene substrates for olefin metathesis reactions, differing in their hydrodynamic radius and polarity, were used to probe the pore confinement effect during the reaction. Our results reveal significant confinement effects, which enhance the macrocyclization over oligomerization selectivity in the pores as compared to the homogeneous reaction. A clear trend between substrate size and MMC:O selectivity was found and can be attributed to a size‐related, slower diffusion of the larger substrates into the pores, thus reducing the efficiency of confinement effects for the larger substrates while enhancing it for the smaller ones. Our results point to the possibility of tailoring the selectivity of olefin metathesis and other size‐sensitive catalytic reactions by adjusting the subtle interplay between the size and polarity of both the COF pores and the substrates used for catalysis.

## Conflict of interest

The authors declare no conflict of interest.

1

## Supporting information

As a service to our authors and readers, this journal provides supporting information supplied by the authors. Such materials are peer reviewed and may be re‐organized for online delivery, but are not copy‐edited or typeset. Technical support issues arising from supporting information (other than missing files) should be addressed to the authors.

Supporting Information

## Data Availability

The data that support the findings of this study are available from the corresponding author upon reasonable request.

## References

[1] M. S. Lohse , T. Bein , Adv. Funct. Mater. 2018, 28, 1705553.

[2] X. Wang , L. Chen , S. Y. Chong , M. A. Little , Y. Wu , W. H. Zhu , R. Clowes , Y. Yan , M. A. Zwijnenburg , R. S. Sprick , A. I. Cooper , Nat. Chem. 2018, 10, 1180–1189. 30275507 10.1038/s41557-018-0141-5

[3] B. P. Biswal , H. A. Vignolo-González , T. Banerjee , L. Grunenberg , G. Savasci , K. Gottschling , J. Nuss , C. Ochsenfeld , B. V. Lotsch , J. Am. Chem. Soc. 2019, 141, 11082–11092. 31260279 10.1021/jacs.9b03243 PMC6646957

[4] L. Ascherl , E. W. Evans , M. Hennemann , D. Di Nuzzo , A. G. Hufnagel , M. Beetz , R. H. Friend , T. Clark , T. Bein , F. Auras , Nat. Commun. 2018, 9, 3802. 30228278 10.1038/s41467-018-06161-w PMC6143592

[5] S. Jhulki , J. Kim , I. C. Hwang , G. Haider , J. Park , J. Y. Park , Y. Lee , W. Hwang , A. A. Dar , B. Dhara , S. H. Lee , J. Kim , J. Y. Koo , M. H. Jo , C. C. Hwang , Y. H. Jung , Y. Park , M. Kataria , Y. F. Chen , S. H. Jhi , M. H. Baik , K. Baek , K. Kim , J. Cleaner Prod. 2020, 6, 2035–2045.

[6] Y. Ge , H. Zhou , Y. Ji , L. Ding , Y. Cheng , R. Wang , S. Yang , Y. Liu , X. Wu , Y. Li , J. Phys. Chem. C 2018, 122, 27495–27506.

[7] Y. Zhi , Z. Wang , H. L. Zhang , Q. Zhang , Small 2020, 16, 2001070. 10.1002/smll.202001070 32419332

[8] S.-Y. Ding , J. Gao , Q. Wang , Y. Zhang , W.-G. Song , C.-Y. Su , W. Wang , J. Am. Chem. Soc. 2011, 133, 19816–19822. 22026454 10.1021/ja206846p

[9] H. Xu , J. Gao , D. Jiang , Nat. Chem. 2015, 7, 905–912. 26492011 10.1038/nchem.2352

[10] Q. Sun , Y. Tang , B. Aguila , S. Wang , F. Xiao , P. K. Thallapally , A. M. Al-Enizi , A. Nafady , S. Ma , Angew. Chem. 2019, 131, 8762–8767; 10.1002/anie.201900029 30957347

[11] J. Guo , D. Jiang , ACS Cent. Sci. 2020, 6, 869–879. 32607434 10.1021/acscentsci.0c00463 PMC7318070

[12] Q. Fang , S. Gu , J. Zheng , Z. Zhuang , S. Qiu , Y. Yan , Q. Fang , S. Gu , J. Zheng , Z. Zhuang , Y. S. Yan , S. Qiu , Angew. Chem. Int. Ed. 2014, 53, 2878–2882; 10.1002/anie.201310500 24604810

[13] B. Mitschke , M. Turberg , B. List , Chem 2020, 6, 2515–2532.

[14] C. Heinis , Nat. Chem. Biol. 2014, 10, 696–698. 25038789 10.1038/nchembio.1605

[15] S. Monfette , D. E. Fogg , Chem. Rev. 2009, 109, 3783–3816. 19537778 10.1021/cr800541y

[16] F. Ziegler , J. Teske , I. Elser , M. Dyballa , W. Frey , H. Kraus , N. Hansen , J. Rybka , U. Tallarek , M. R. Buchmeiser , J. Am. Chem. Soc. 2019, 141, 19014–19022. 31694374 10.1021/jacs.9b08776

[17] J. E. Jee , J. L. Cheong , J. Lim , C. Chen , S. H. Hong , S. S. Lee , J. Org. Chem. 2013, 78, 3048–3056. 23432508 10.1021/jo302823w

[18] S. T. Emmerling , R. Schuldt , S. Bette , L. Yao , J. Kästner , ChemRxiv. Cambridge Open Engag. 2021, 10.33774/chemrxiv-2021-lszg0.

[19] H. M. Rietveld , J. Appl. Crystallogr. 1969, 2, 65–71.

[20] M. A. Gotthardt , R. Schoch , S. Wolf , M. Bauer , W. Kleist , Dalton Trans. 2015, 44, 2052–2056. 25518915 10.1039/c4dt02491e

[21] M. Benedikter , J. Musso , M. K. Kesharwani , K. Leonard Sterz , I. Elser , F. Ziegler , F. Fischer , B. Plietker , W. Frey , J. Kastner , M. Winkler , J. van Slageren , M. Nowakowski , M. Bauer , M. R. Buchmeiser , ACS Catal. 2020, 10, 14810–14823.

[22] U. Tallarek , J. Hochstrasser , F. Ziegler , X. Huang , C. Kübel , M. R. Buchmeiser , ChemCatChem 2021, 13, 281–292.

[23] F. Ziegler , T. Roider , M. Pyschik , C. P. Haas , D. Wang , U. Tallarek , M. R. Buchmeiser , ChemCatChem 2021, 13, 2234–2241.

