# Supplementary material for: Olefin Metathesis in Confinement: Towards Covalent Organic Framework Scaffolds for Increased Macrocyclization Selectivity
Source: Chemistry. 2022 Jan 5;28(8):e202104108. doi: 10.1002/chem.202104108 (PMC9305778; doi:10.1002/chem.202104108)
Supplement: Supplementary file 1 — Supporting Information [file CHEM-28-0-s001.pdf]

Chemistry–A European Journal

Supporting Information

Olefin Metathesis in Confinement: Towards Covalent Organic Framework Scaffolds for Increased Macrocyclization Selectivity

Sebastian T. Emmerling, Felix Ziegler, Felix R. Fischer, Roland Schoch, Matthias Bauer, Bernd Plietker, Michael R. Buchmeiser, and Bettina V. Lotsch*

Table of Contents

Table of Contents 1

Experimental Procedures 2

S1 Materials and Methods 2

S2 Experimental Section 3

S3 FT-IR spectroscopy 12

S4 XRPD measurements and refinements 13

S5 Gas Sorption Experiments 19

S6 Solid State NMR 22

S7 XAS 23

S8 Liquid State NMR 31

S9 SEM/TEM and EDX analysis 35

S10 TGA analysis 37

S11 References 38

Experimental Procedures

S1 Materials and Methods

Chemicals. All catalytic reactions were performed under the exclusion of air and moisture in a N_2-filled glove box (MBraun Labmaster) unless noted otherwise; all COF building block reactions were carried out under Ar *via* Schlenk technique unless noted otherwise. Chemicals were purchased from *ABCR*, *Acros Organics*, *Alfa Aesar*, *Sigma Aldrich, Fluka* and *TCI*. 1,2-Dichlorobenzene (anhydrous) was purchased from Sigma Aldrich. *n*-Pentane was dried using an MBraun SPS-800 solvent purification system and stored over 4 Å molecular sieves. Deuterated solvents were stored over activated alumina and 4 Å molecular sieves for a minimum of 24 h prior to use. All other solvents, unless otherwise specified, were obtained from Acros Organics, and used without further purification. 4,4'-Dibromo-[1,1'-biphenyl]-2-amine (**6**) was synthesized according to a literature procedure.[1]

X-Ray Powder Diffraction. XRPD patterns were collected on a laboratory powder diffractometer in Debye-Scherrer geometry (Stadi P-diffractometer (Stoe), using Co-$K_{\alpha1}$ radiation from a primary Ge(111)-Johann-type monochromator and a Mythen 1 K detector (Dectris)). The sample was sealed in a 1.0 mm diameter borosilicate glass capillary, which was spun during measurements. Each powder pattern was collected in a 2θ range from 0 ° to 40 ° with a total scan time of 5 hours. The program TOPAS 6.0 was used for the data analyses. The initially model for refinement was build and geometrically optimized in BIOVIA Materials Studio 2017 (17.1.0.48). Single atom positins were not refined. The LP factor was adjusted for the device and fixed at 31.787, in a Double-Voigt approach crystallite size and strain was accounted for and a stephens model (hexagonal) was used.

FT-IR Spectroscopy. Infrared spectra were measured in attenuated total reflection (ATR) geometry on a PerkinElmer UATR Two equipped with a diamond crystal. All spectra were background corrected.

Liquid State Nuclear Magnetic Resonance. Liquid state nuclear magnetic resonance (NMR) measurements for the COF building blocks were performed on a JEOL ECZ 400S 400 MHz spectrometer (magnetic field 9.4 T). ^{1}H, ^{13}C and ^{15}N measurements were performed in 5 mm NMR tubes using deuterium field lock. NMR spectra for the Ru-catalysis reactions were recorded on a Bruker Avance III 400 spectrometer. NMR spectra were internally calibrated to the corresponding solvent signal.[2] Abbreviations for multiplicities: s (singlet), bs (broad singlet), d (dublet), t (triplet), q (quartet), hept (heptet), m (multiplet).

Solid State Nuclear Magnetic Resonance. Solid-state NMR (ssNMR) was recorded on a Bruker Avance III 400 MHz spectrometer (magnetic field 9.4 T). Samples were packed in 4 mm ZrO_2 rotors, which were spun at 12-14 kHz in a Bruker WVT BL4 double resonance MAS probe. Chemical shift was referenced relative to tetramethylsilane (^{13}C). A standard cross-polarization sequence with a 2 ms ramped contact pulse was used for ^{13}C and a total of 4096-8192 scans were routinely accumulated. All spectra were background corrected.

Sorption Measurements. Sorption measurements were performed on a Quantachrome Instruments Autosorb iQ 3 with nitrogen at 77 K. The pore size distribution (PSD) was determined from nitrogen adsorption or desorption isotherms using the QSDFT (cylindrical pores, adsorption branch) kernel in carbon for nitrogen at 77 K implemented in the ASiQwin software v 3.01. Samples were activated under high vacuum at room temperature for 12 h before measurement.

Mass Spectrometry
Electrospray ionization (ESI) mass spectrometry was performed on a Thermo Finnigan LTQ FT in the positive and negative mode. Samples were dissolved in an acetonitrile/water mixture.

Inductively-Coupled Plasma Optical Emission Spectroscopy (ICP-OES) was performed on a VARIA VISTA RL simultaneous spectrometer (Agilent Technologies, Santa Clara, California, USA) with a CCD-detector. Samples were dissolved in conc. nitric acid (65%) at 185 °C for 25 min using a Discover-SP-D microwave by CEM.

Scanning Electron Microscopy. SEM SE (secondaryelectron) detector images were obtained on a Zeiss Merlin.

Transmission Electron Microscopy. TEM was performed with a Philips CM30 ST at 300kV (LaB_6cathode). Samples were prepared dry onto a holey carbon/copper grid.

Thermal Analysis. Thermogravimetric analysis was performed on a *NETZSCH STA 449 F3 Jupiter*. Measurement was carried out with 5 mg of sample in an Al_2O_3 crucibles under Helium protective gas flow in a temperature range between 40 to 1000 °C and a heating rate of 10 K/min.

Results and Discussion

S2 Experimental Section

COF Synthesis

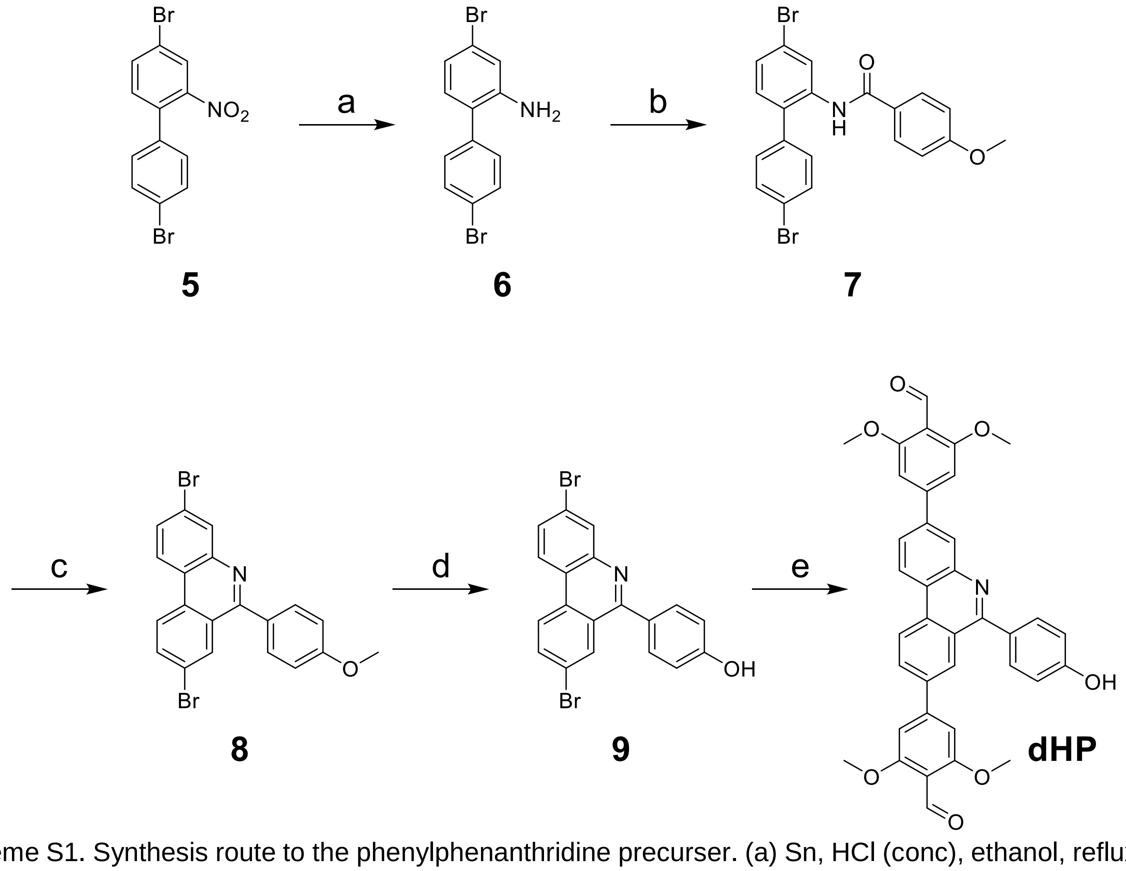

Scheme S1. Synthesis route to the phenylphenanthridine precurser. (a) Sn, HCl (conc), ethanol, reflux, 5h; (b) 4-methoxybenzoyl chloride, Et_3N, CH_2Cl_2, rt, 1h; (c) 2-chloropyridine, trifluormethansulfonic anhydride, CH_2Cl_2, 0 °C to 140 °C, 0.5 h; (d) BF_3•Me_2S, CH_2Cl_2, rt, 16 h; (e) 4-formyl-3,5-dimethoxyphenylboronic acid, tetrakis(triphenylphosphin)palladium(0), Na_2CO_3, 1,4-dioxane, 90 °C, 72 h.

Synthesis of *N*-(4,4'-dibromo-[1,1'-biphenyl]-2-yl)-4-methoxybenzamide (7). *N*-(4,4'-dibromo-[1,1'-biphenyl]-2-yl)-4-methoxybenzamide (**7**) was synthesized according to a modified literature procedure.[3] Under argon **6** (1.00 g, 3.06 mmol) and triethylamine (1.28 mL, 9.18 mml) were dissolved in dry CH_2Cl_2

(35 mL) and 4-methoxybenzoyl chloride (0.62 mL, 4.59 mmol) was added dropwise. The reaction mixture was stirred at room temperature overnight for 16 h, then ethanol (5 mL) was added. After 1 h, the solution was poured into water (50 mL) and extracted with CH_2Cl_2. The combined organic layers were dried over anhydrous Na_2SO_4 and evaporated under reduced pressure. The resulting crude product was purified by recrystallization from CH_2Cl_2 /*n*-hexane to obtain **7** (1.25 g, 2.71 mmol, 88%). ^{1}H NMR (400 MHz, $CDCl_3$) δ 8.70 (d, J = 2.0 Hz, 1H), 7.78 (s, 1H), 7.67 – 7.61 (m, 2H), 7.58 – 7.53 (m, 2H), 7.32 (dd, J = 8.2, 2.0 Hz, 1H), 7.30 – 7.26 (m, 2H), 7.10 (d, J = 8.1 Hz, 1H), 6.92 – 6.88 (m, 2H), 3.84 (s, 3H) ppm. ^{13}C NMR (101 MHz, $CDCl_3$) δ 164.7, 162.8, 136.3, 136.2, 132.7, 131.2, 130.9, 129.9, 128.9, 127.5, 126.4, 124.5, 122.9, 122.8, 114.8, 55.6 ppm. FT-IR v_{max}/cm^{-1} 3434, 2839, 1669, 1574, 1503, 1412, 1252, 1179, 1031, 845, 808, 754, 542. HRMS (ESI) exact mass calculated for [M+H]+ ($C_{20}H_{15}Br_2NO_2$) requires m/z 461.9449, found m/z 461.95227.

Synthesis of 3,8-dibromo-6-(4-methoxyphenyl)phenanthridine (8). 3,8-dibromo-6-(4-methoxyphenyl)phenanthridine (**8**) was synthesized according to a modified literature method.[4] Under argon a *Biotage* microwave vial was charged with **7** (1.20 g, 2.6 mmol), 2-chloropyridine (0.29 mL, 3.12 mmol) and dry CH_2Cl_2 (13 mL). Trifluormethansulfonic anhydride (0.48 mL, 2.86 mmol) was added dropwise at 0°C. After 5 minutes, the solution was warmed to room temperature, the vial was capped and under microwave irradiation, the solution was heated to 140 °C for 30 minutes. After the solution was cooled to room temperature, triethylamine (0.73 mL, 5.2 mmol) was added dropwise to neutralize the trifluormethanesulfonate salts. All volatiles were removed under reduced pressure and the residue was purified by flash column chromatography (SiO_2, *n*-hexane/ CH_2Cl_2) to obtain **4** (1.06 g, 2.39 mmol, 92%). ^{1}H NMR (400 MHz, $CDCl_3$) δ 8.46 (d, J = 8.8 Hz, 1H), 8.41 – 8.33 (m, 2H), 8.30 (d, J = 2.0 Hz, 1H), 7.92 (dd, J = 8.8, 2.1 Hz, 1H), 7.74 (dd, J = 8.7, 2.1 Hz, 1H), 7.70 – 7.64 (m, 2H), 7.15 – 7.08 (m, 2H), 3.93 (s, 3H) ppm. ^{13}C NMR (101 MHz, $CDCl_3$) δ 160.9, 160.7, 144.8, 134.2, 132.9, 132.0, 131.5, 131.3, 130.4, 126.7, 124.1, 123.4, 123.1, 121.9, 121.7, 114.3, 55.6 ppm. FT-IR v_{max}/cm^{-1} 2958, 2839, 1593, 1557, 1511, 1463, 1357, 1303, 1249, 1176, 1034, 964, 828, 799, 596, 518. HRMS (ESI) exact mass calculated for [M+H]+ ($C_{20}H_{13}Br_2NO$) requires m/z 443.9343, found m/z 443.94166.

Synthesis of 4-(3,8-dibromophenanthridin-6-yl)phenol (9). Under argon a flask was charged with **3** (0.50 g, 1.13 mmol), and dry CH_2Cl_2 (30 mL). A boron trifluoride-dimethyl sulfide complex (1.42 mL, 13.6 mmol) was added dropwise at room temperature. The solution was stirred at room temperature for 16 h, then cooled to 0 °C and methanol was added dropwise to quench the reaction. All volatiles were removed under reduced pressure and the residue was purified by flash column chromatography (SiO_2, CH_2Cl_2/MeOH) to obtain **9** (0.42 g, 0.98 mmol, 87%). ^{1}H NMR (400 MHz, DMSO-D6) δ 8.86 (d, J = 9.6 Hz, 1H), 8.76 (d, J = 9.0 Hz, 1H), 8.27 (d, J = 2.0 Hz, 1H), 8.19 (d, J = 7.9 Hz, 2H), 7.92 (dd, J = 8.8, 2.1 Hz, 1H), 7.67 – 7.59 (m, 2H), 7.10 – 7.00 (m, 2H), 5.74 (s, 1H) ppm. ^{13}C NMR (101 MHz, DMSO-D6) δ 160.4, 159.7, 135.8, 132.2, 131.9, 131.3, 130.9, 129.0, 125.7, 125.6, 125.2, 123.1, 121.9, 115.6 ppm. FT-IR v_{max}/cm^{-1} 3368 (br), 3182, 3093, 1636, 1582, 1468, 1360, 1225, 1184, 1084, 1068, 994, 818, 661, 520. HRMS (ESI) exact mass calculated for [M+H]+ ($C_{19}H_{11}Br_2NO$) requires m/z 429.9187, found m/z 429.92596.

Synthesis of 4,4'-(6-phenylphenanthridine-3,8-diyl)bis(2,6-dimethoxybenzaldeyde) (dHP). Under argon in a 20 mL *Biotage* microwave vial, **9** (0.25 g, 0.58 mmol), 4-formyl-3,5-dimethoxyphenylboronic acid (0.49 g, 2.34 mmol) and tetrakis(triphenylphosphin)palladium(0) (0.068 g, 0.06 mmol) were dissolved in 1,4-dioxane (15 mL) and 2 M aqueous Na_2CO_3 (2.6 mL) was added. After degassing the solution with argon for 30 minutes the vial was capped and the mixture was stirred for 72 h at 90 °C. After cooling to room temperature, all solvents were removed and the residual solid was extracted with $CHCl_3$ (3 x 50 mL). The combined organic extracts were evaporated under reduced pressure. The solid residue was purified by flash column chromatography (SiO_2, *n*-hexane/$CHCl_3$ with 1% Et_3N) to obtain **dHP** (0.21 g, 0.35 mmol, 61%). ^{1}H NMR (400 MHz, CHLOROFORM-D) δ 10.53 (s, 1H), 10.45 (s, 1H), 8.84 (d, J = 8.7 Hz, 1H), 8.73 (d, J = 8.7 Hz, 1H), 8.40 (s, 1H), 8.22 (d, J = 8.3 Hz, 1H), 8.08 (d, J = 8.6 Hz, 1H), 7.90 (d, J = 4.9 Hz, 2H), 7.66 – 7.61 (m, 3H), 6.98 (s, 2H), 6.79 (s, 2H), 3.99 (s, 6H), 3.96 (s, 6H) ppm. ^{13}C NMR (101 MHz, CHLOROFORM-D) δ 188.96, 162.63, 130.27, 130.24, 130.21, 130.17, 128.83, 123.62, 123.47, 123.09, 113.68, 113.57, 103.32, 103.18, 56.40, 56.30 ppm. FT-IR v_{max}/cm^{-1} 3423 (br), 2936, 2876, 1676, 1597, 1567, 1455, 1393, 1241, 1123, 809, 705, 576. HRMS (ESI) exact mass calculated for [M+H]+ ($C_{37}H_{29}NO_7$) requires m/z 600.1944, found m/z 600.20211.

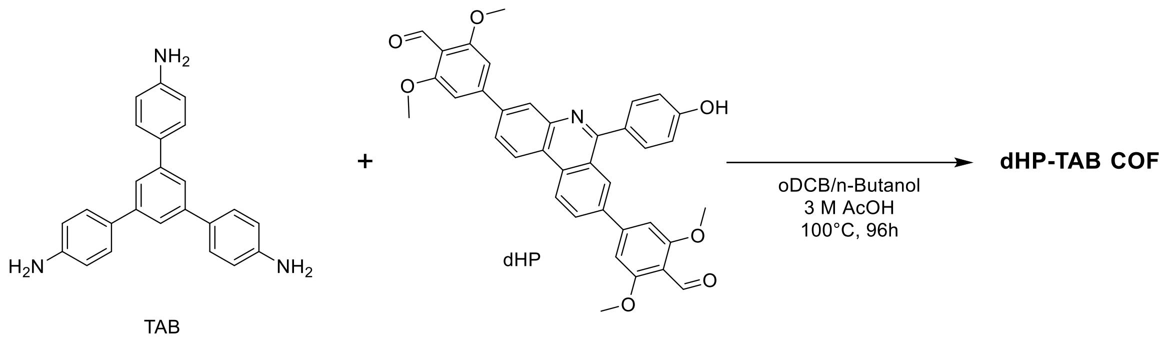

Scheme 2. Synthesis of dHP-COF.

Synthesis of dHP-TAB COF. 1,3,5-Tris(4-aminophenyl)benzene (**TAB)** (0.1 mmol, 35.1 mg) and **dHP** (0.15 mmol, 90.0 mg) were placed into a 5 mL *Biotage* microwave vial. 4 mL of a 1,2-dichlorobenzene/*n*-butanol (1:4) mixture were added, followed by 80 µL 3M AcOH. The vial was capped and placed in an aluminum heating block that was preheated to 100°C. Under stirring at 500 rpm the mixture was kept at 100°C for 96 h, then it was allowed to cool to room temperature. The solid was filtered off, washed with MeOH and then subjected to Soxhlet extraction with MeOH for 16 h. The MeOH soaked solid was then activated by scCO2 drying and further under high vacuum for 24 h to obtain 93.1 mg **dHP-TAB** COF (78 %).

Substrates and Ru-catalyst

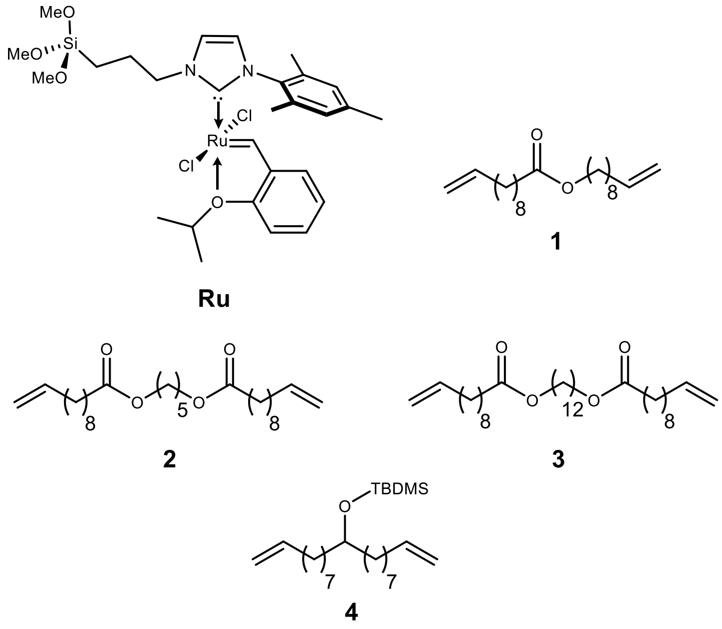

Scheme 3. Ru-catalyst and substrated used in macrocyclization reactions.

RuCl$_2$(N-mesityl-N-(3-(trimethoxysilyl)prop-1-yl)-imidazol-2-ylidene)(CH-2-(2-PrO-C$_6$H$_4$)) **Ru,**[5] dec-9-en-1-yl undec-10-enoate **1**,[5] pentane-1,5-diyl bis(undec-10-enoate) **2**,[5,6] dodecane-1,12-diyl bis(undec-10-enoate) **3**,[5] *tert*-butyldimethyl(nonadeca-1,18-dien-10-yloxy)silane **4**,[5,6] (E)+(Z)-oxacycloicos-11-en-2-one,[6] (E)+(Z)-1,7-dioxacycloheptacos-17-ene-8,27-dione,[7] (E)+(Z)-1,14-dioxacyclotetratriacont-24-ene-15,34-dione[5] and (E)+(Z)-*tert*-butyl(cycloheptadec-9-en-1-yloxy)dimethylsilane[6] were synthesized according to the literature.

Immobilization of Ru and Ru@dHP-TAB loading

A solution of **Ru** (10 mg) in 1,2-dichlorobenzene was added to the **dHP-TAB COF** (~100 mg). For the removal of nitrogen in the pores, vacuum was applied. The suspension was stirred for 16 hours at room temperature. Then, the suspension was filtered, and the resulting **Ru@dHP-TAB COF** containing the immobilized catalyst was washed with 1,2-dichlorobenzene (200 mL), *n*-pentane (200 mL), dried in vacuo at room temperature for 3 h, and stored under an inert atmosphere at −35 °C.

Table S1: Ru-content of COF as determined by ICP-OES.

Material	*c*(Ru) on COF [µmol Ru/g COF]
**Ru@dHP-TAB**	**42.3**

Leak test of Ru@dHP-TAB

Ru@dHP-TAB (30 mg) was stirred in 4 mL benzene at 50 °C for 16 h. The solid was filtered off, the solvent removed and the residue was dired under vacuum at elevated temperature. The remaining oil-like residue was tested by ICP-OES for its ruthenium content. No ruthenium was detected.

Additionally, **Ru@dHP-TAB** was tested by ICP-OES after performing the general procedure for the RCM of **α,ω**-dienes with substrate **1.** The recovered material showed a concentration c(Ru) of 51.74 µmol Ru/g COF, even slighty higher than the pristine material. We attribute the increase to slight sample inhomegeneities in the batch used.

Macrocyclization Reactions

General procedure for the RCM of α,ω-dienes (GP-1). The substrate was dissolved in C_6D_6 (1000 µL) and the catalyst (stock solution) was added. After 16 hours, ^1H NMR spectra were acquired. Conversion and macro(mono)cyclization:oligomer (MMC:O) ratios were determined by integration of the corresponding signals.

***Example reaction with 0.5 mol% catalyst, MMC with* Ru: 1** (8.1 mg, 0.025 mmol, 1 eq.) was dissolved in C_6D_6 (995.8 µL, 25.1 mM) and a stock solution of catalyst **Ru** (4.2 µL, 2.9 mM; 0.5 mol-%) was added. The resulting mixture (substrate concentration 25 mM) was stirred for 16 hours at 50 °C and subjected to ^1H NMR spectroscopy. Overall conversion: 81%. MMC:O = 0.9 (selectivity = 47%).

For the correct determination of the conversion by NMR, the deconvolution function of MestReNova (Version 12.0.0) was used. This was necessary because part of the oligomer signals of the terminal double bonds were covered by the signals of the starting material (see Figure S1).

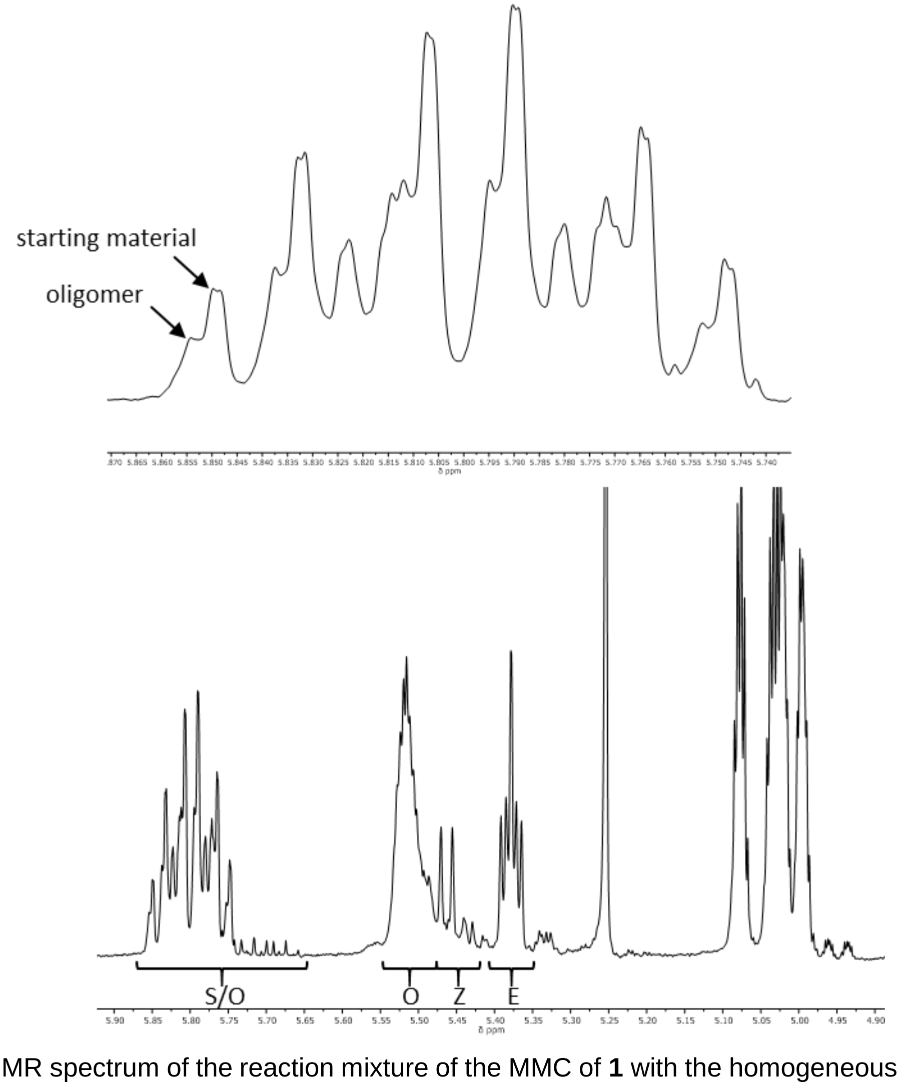

Figure S1: ^1H NMR spectrum of the reaction mixture of the MMC of **1** with the homogeneous catalyst **Ru** in C_6D_6. MMC:O = 0.9 (selectivity = 47%) and an enlarged area where deconvolution was applied.

Table S2: Conversion, MMC:O ratio and selectivity for the RCM of substrate **1 - 4** by the action of **Ru** (0.5 mol-%) as determined by NMR.

Substrate	Conversion [%]	MMC:O	Selectivity [%]
**1**	81	0.90	47
**2**	77	0.84	46
**3**	80	0.65	39
**4**	73	0.40	28

General procedure for the RCM of α,ω-dienes with Ru@dHP-TAB (GP-2). The substrate was dissolved in C_6D_6 (1.0 mL) and the corresponding COF material containing the Ru-catalyst (3-6 mg, depending on the Ru content and the catalyst:substrate ratio) was added. After 16 hours, ^1H NMR data were acquired. Conversion, the MMC:O ratio and the selectivity were determined by integration of the corresponding signals.

***MMC with* Ru@dHP-TAB: 1** (8.1 mg, 0.025 mmol, 1 eq.) was dissolved in C_6D_6 (1.00 mL, 25 mM) and **Ru@dHP-TAB** (2.9 mg, 0.5 mol-%) was added. The resulting mixture was stirred at 50 °C for 16 hours and subjected to ^1H NMR spectroscopy. Overall conversion: 11%. MMC:O = 1.3 (selectivity = 56%).

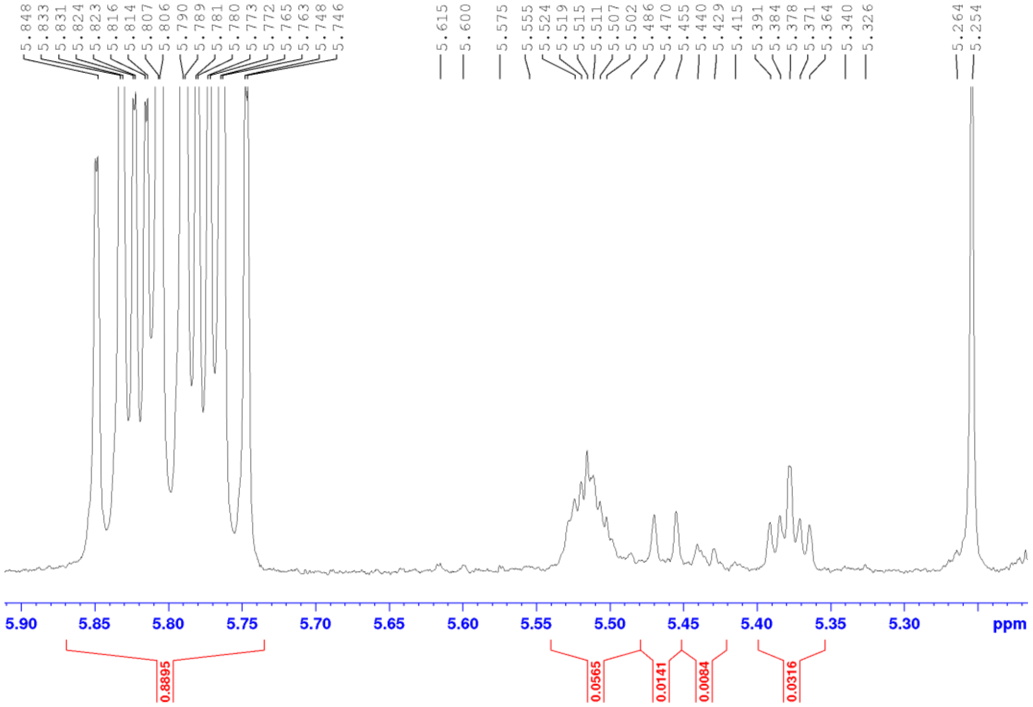

Figure S2: ^1H NMR spectrum of the MMC reaction mixture of **1** with the homogeneous catalyst **Ru@dHP-TAB** in C_6D_6. MMC:O = 1.3 (selectivity = 56%).

***MMC with* Ru@dHP-TAB: 1** (8.1 mg, 0.025 mmol, 1 eq.) was dissolved in C_6D_6 (1.00 mL, 25 mM) and **Ru@dHP-TAB** (5.8 mg, 1.0 mol-%) was added. The resulting mixture was stirred at 50 °C for 16 hours and subjected to ^1H NMR spectroscopy. Overall conversion: 15%. MMC:O = 1.3 (selectivity = 56%).

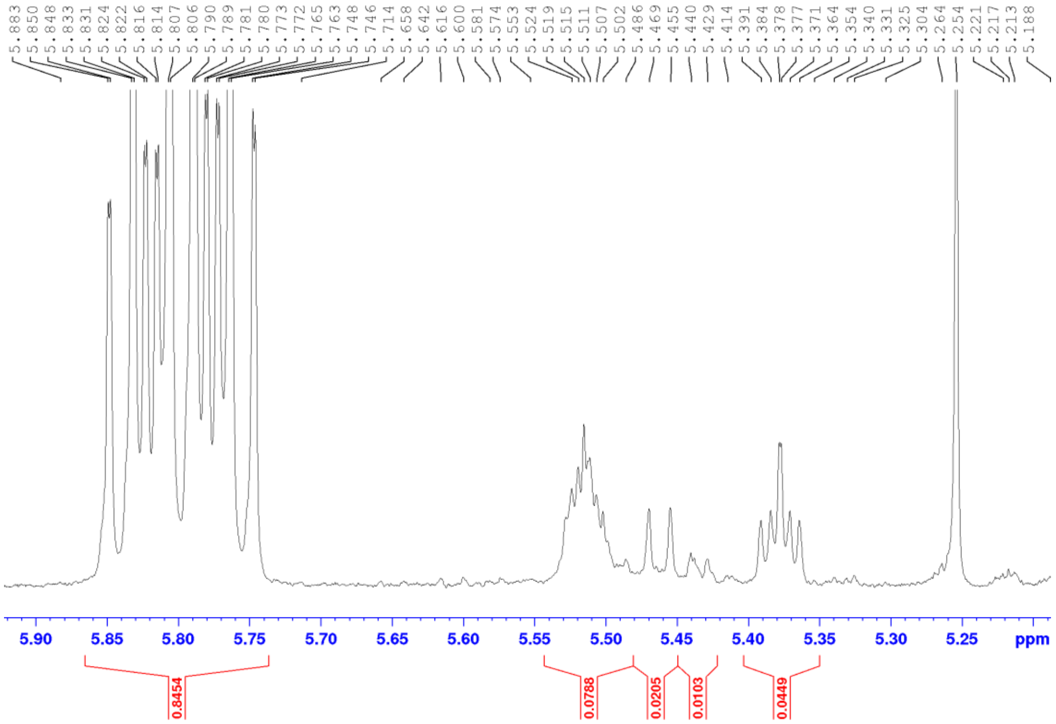

Figure S3: ^{1}H NMR spectrum of the MMC reaction mixture of **1** with the homogeneous catalyst **Ru@dHP-TAB** in C_6D_6. MMC:O = 1.3 (selectivity = 56%).

***MMC with* Ru@dHP-TAB: 2** (10.3 mg, 0.025 mmol, 1 eq.) was dissolved in C_6D_6 (1.00 mL, 25 mM) and **Ru@dHP-TAB** (2.9 mg, 0.5 mol-%) was added. The resulting mixture was stirred at 50 °C for 16 hours and subjected to ^{1}H NMR spectroscopy. Overall conversion: 11%. MMC:O = 1.0 (selectivity = 49%).

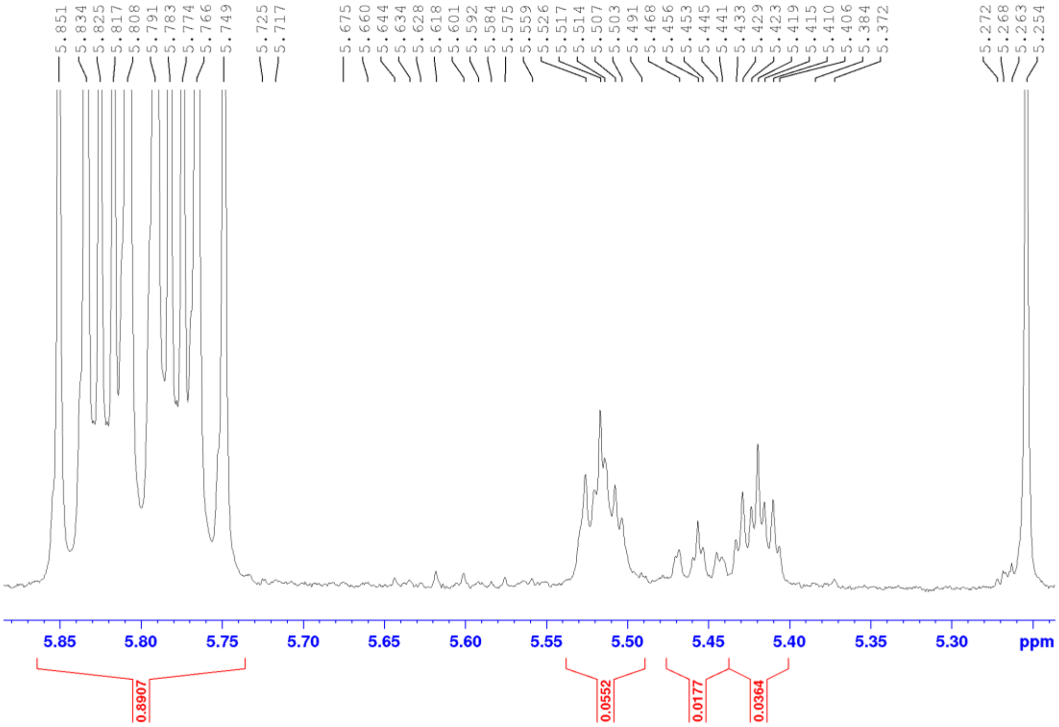

Figure S4: ^{1}H NMR spectrum of the MMC reaction mixture of **2** with the homogeneous catalyst **Ru@dHP-TAB** in C_6D_6. MMC:O = 1.0 (selectivity = 49%).

***MMC with* Ru@dHP-TAB: 3** (13.4 mg, 0.025 mmol, 1 eq.) was dissolved in C_6D_6 (1.00 mL, 25 mM) and **Ru@dHP-TAB** (2.9 mg, 0.5 mol-%) was added. The resulting mixture was stirred at 50 °C for 16 hours and subjected to ^{1}H NMR spectroscopy. Overall conversion: 11%. MMC:O = 0.6 (selectivity = 39%).

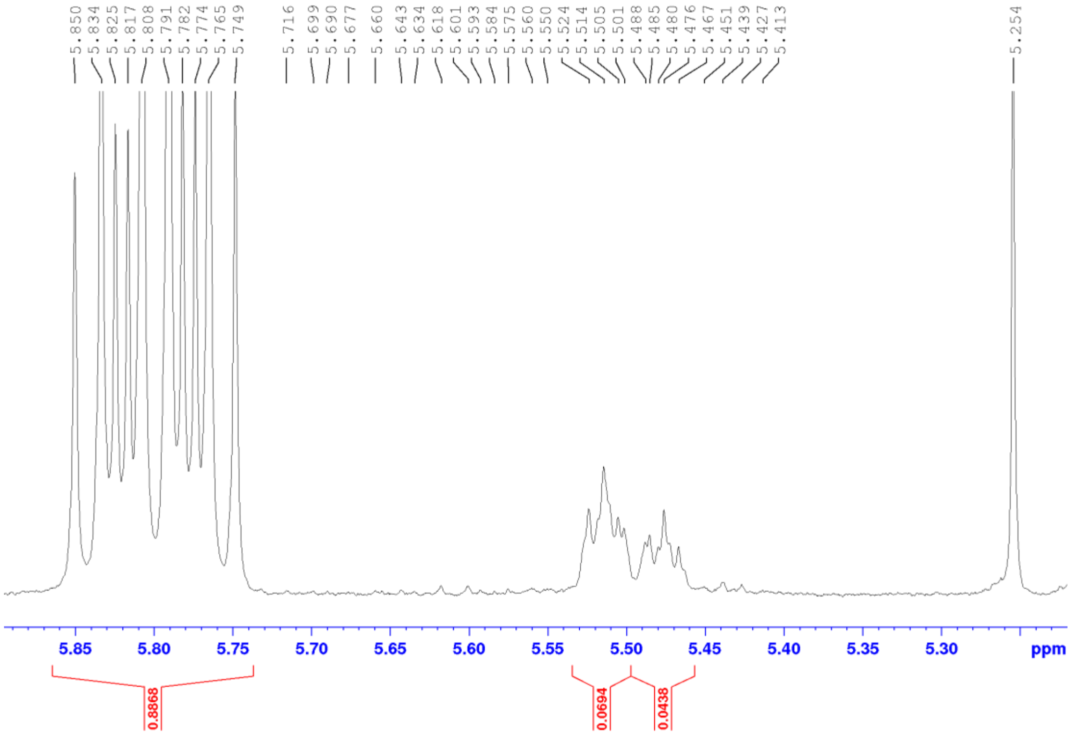

Figure S5: ^{1}H NMR spectrum of the MMC reaction mixture of **3** with the homogeneous catalyst **Ru@dHP-TAB** in C_6D_6. MMC:O = 0.6 (selectivity = 39%).

***MMC with* Ru@dHP-TAB: 4** (9.9 mg, 0.025 mmol, 1 eq.) was dissolved in C_6D_6 (1.00 mL, 25 mM) and **Ru@dHP-TAB** (2.9 mg, 0.5 mol-%) was added. The resulting mixture was stirred at 50 °C for 16 hours and subjected to ^{1}H NMR spectroscopy. Overall conversion: 12%. MMC:O = 0.5 (selectivity = 35%).

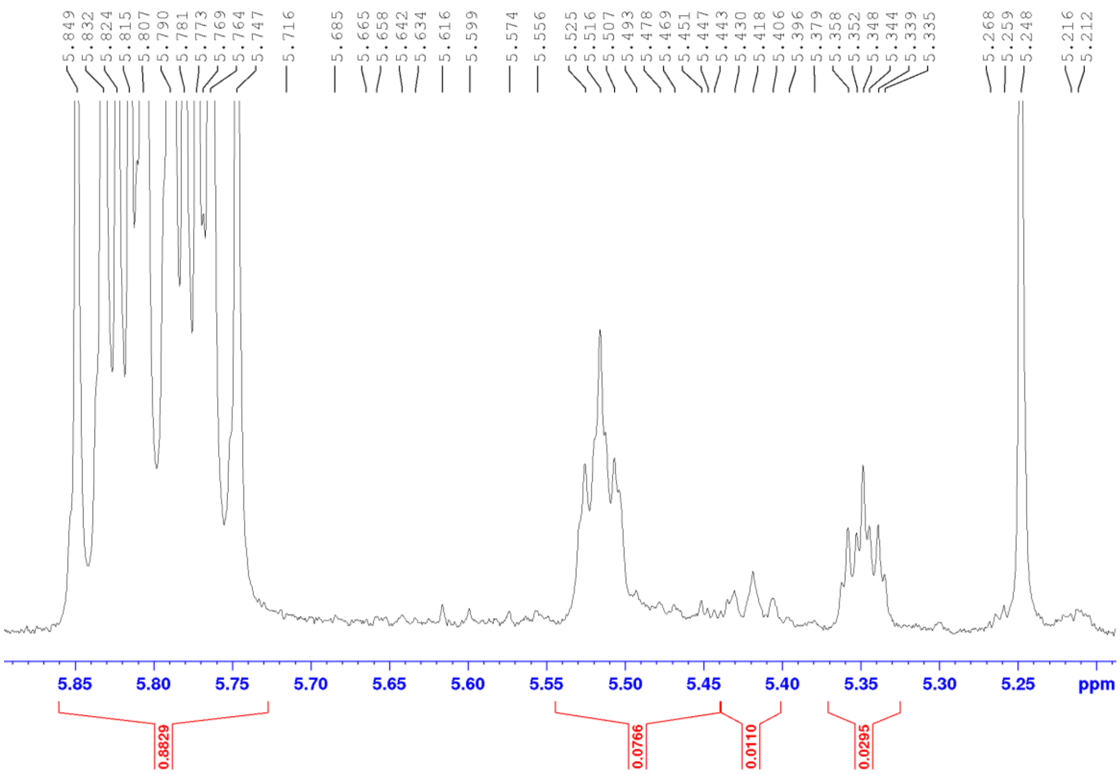

Figure S6: ^{1}H NMR spectrum of the MMC reaction mixture of **4** with the homogeneous catalyst **Ru@dHP-TAB** in C_6D_6. MMC:O = 0.5 (selectivity = 35%).

Kinetics

Each table entry is an independent reaction which was hot filtered at the time mentioned in Table S3. The NMR solution shows no change over time.

Table S3: Conversion, MMC:O ratio and selectivity for the RCM of substrate **1** by the action of **Ru@dHP-TAB** terminated at a different time.

Time [min]	Conversion [%]	MMC:O	Selectivity [%]
30	3	1.1	52
60	4	1.1	52
180	7	1.3	57
360	9	1.2	55
960	11	1.3	56

S3 FT-IR spectroscopy

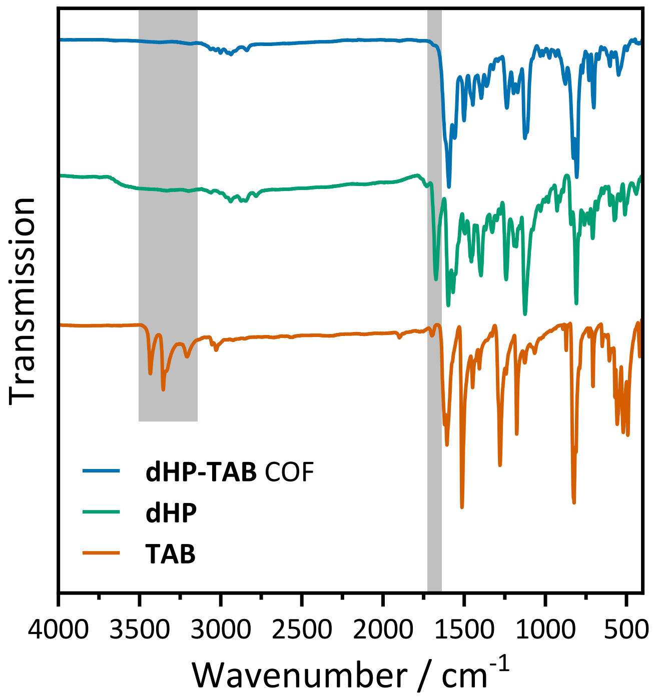

Figure S7. FT-IR comparison of **dHP-TAB COF** and the starting materials **dHP** and **TAB**.

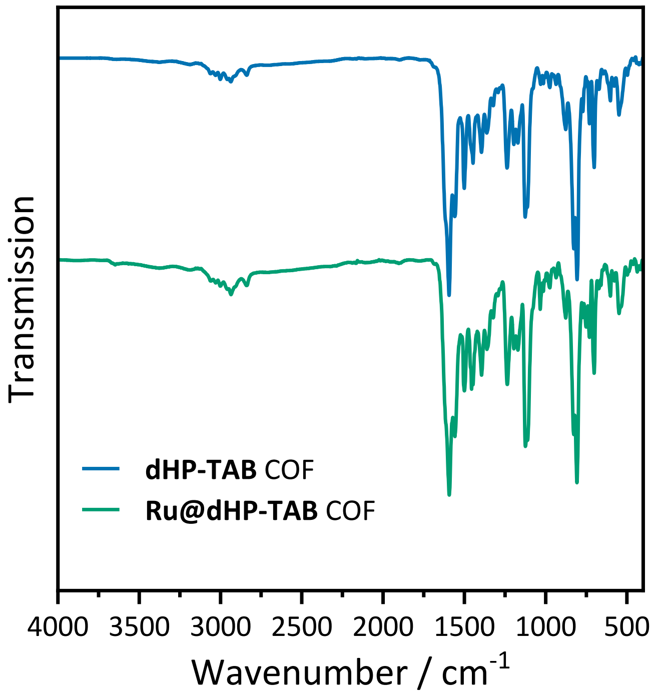

Figure S8. FT-IR comparison of **dHP-TAB COF** and **Ru@dHP-TAB COF.**

S4 XRPD measurements and refinements

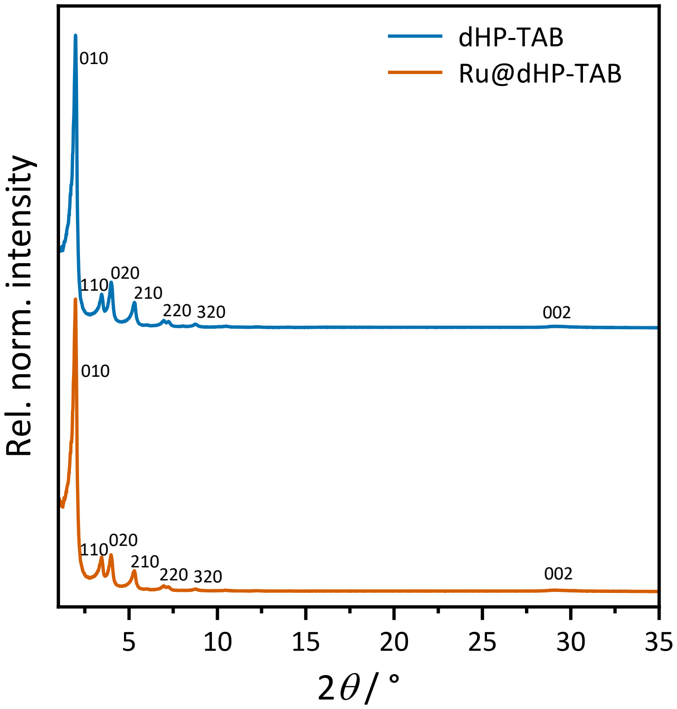

Figure S9. XRPD comparison (λ = 1.789 Å) of **dHP-TAB** COF and **Ru@dHP-TAB** COF**.**

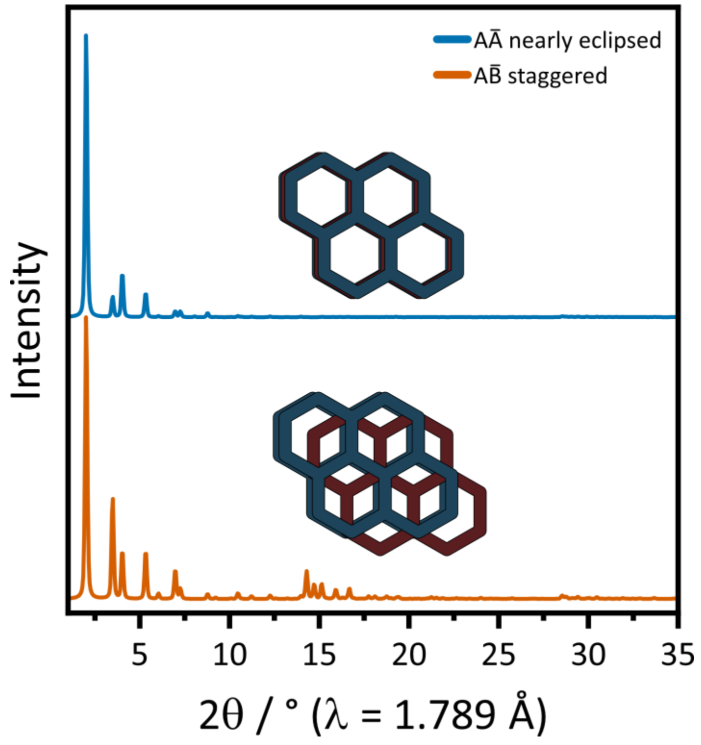

Figure S10. Simulated patterns of AĀ (top) and AB̄ (bottom) stacking for **dHP-TAB**.

Table S4. Results of Rietveld refinement, atom site positions of **dHP-TAB COF**. Atom position were not refined.

*P*31*c*			*a* = *b* = 58.824(10) Å and *c* = 7.3(0) Å		
atom_site_label	_atom_site_fract_x	atom_site_fract_y	atom_site_fract_z	atom_site_type_symbol	atom_site_occupancy
C1	0.05439	0.49425	-0.18419	C	1
C2	0.02761	0.48209	-0.18561	C	1
C3	0.01474	0.49505	-0.12657	C	1
C4	0.02962	0.52109	-0.07024	C	1
C5	0.05657	0.53248	-0.0601	C	1
C6	0.06914	0.51943	-0.11904	C	1
C7	0.98596	0.48225	-0.12263	C	1
C8	0.9747	0.49666	-0.06227	C	1
N9	0.98959	0.52152	-0.01218	N	1
C10	0.0171	0.53487	-0.02117	C	1
C11	0.96958	0.45645	-0.17515	C	1

C12	0.94287	0.44573	-0.17248	C	1
C13	0.93175	0.46035	-0.1168	C	1
C14	0.94797	0.48574	-0.05847	C	1
C15	0.09765	0.53216	-0.10987	C	1
C16	0.10959	0.51828	-0.05672	C	1
C17	0.13661	0.53012	-0.04681	C	1
C18	0.15182	0.55663	-0.08696	C	1
C19	0.13991	0.57098	-0.1388	C	1
C20	0.11286	0.55832	-0.1517	C	1
C21	0.18029	0.56889	-0.07376	C	1
O22	0.15566	0.59758	-0.17398	O	1
C23	0.14391	0.6125	-0.21847	C	1
O24	0.14929	0.51641	-2.40E-04	O	1
C25	0.135	0.48893	-0.00352	C	1
C26	0.03083	0.56315	0.0099	C	1
C27	0.05034	0.58021	-0.10877	C	1
C28	0.06306	0.60672	-0.07743	C	1
C29	0.05603	0.61669	0.06939	C	1
C30	0.03572	0.59989	0.18299	C	1
C31	0.02302	0.57338	0.15161	C	1
C32	0.54621	0.09658	-0.12705	C	1
C33	0.57181	0.10651	-0.1814	C	1
C34	0.58897	0.13322	-0.19012	C	1
C35	0.57995	0.15036	-0.14882	C	1
C36	0.55393	0.14053	-0.09562	C	1
C37	0.53745	0.11363	-0.08449	C	1
C38	0.59839	0.17857	-0.16036	C	1
O39	0.61511	0.14365	-0.23909	O	1
C40	0.62609	0.1273	-0.2345	C	1
O41	0.5456	0.15814	-0.05175	O	1
C42	0.51904	0.14867	-0.01409	C	1
N43	0.8047	0.39561	-0.13729	N	1
C44	0.68131	0.36016	-0.15619	C	1
C45	0.6518	0.30609	-0.15604	C	1

C46	0.72271	0.35881	-0.15447	C	1
C47	0.7355	0.3468	-0.07659	C	1
C48	0.76233	0.35904	-0.07557	C	1
C49	0.77718	0.38382	-0.14637	C	1
C50	0.76469	0.39596	-0.22564	C	1
C51	0.73782	0.3835	-0.23087	C	1
N52	0.40883	0.60416	-0.14723	N	1
C53	0.36013	0.6816	-0.15351	C	1
C54	0.30613	0.6515	-0.15331	C	1
C55	0.36451	0.6419	-0.1515	C	1
C56	0.35526	0.61729	-0.22809	C	1
C57	0.37004	0.60526	-0.22317	C	1
C58	0.39461	0.61758	-0.14594	C	1
C59	0.40423	0.64231	-0.07146	C	1
C60	0.38923	0.65413	-0.07242	C	1
H61	0.06359	0.48387	-0.23435	H	1
H62	0.01743	0.4626	-0.23532	H	1
H63	0.06798	0.55127	3.90E-04	H	1
H64	0.97686	0.44428	-0.22165	H	1
H65	0.93098	0.42619	-0.22051	H	1
H66	0.9399	0.49734	-0.01242	H	1
H67	0.09758	0.49827	-0.02074	H	1
H68	0.10333	0.5686	-0.19602	H	1
H69	0.18827	0.55879	-0.00209	H	1
H70	0.05576	0.57307	-0.22564	H	1
H71	0.07837	0.61955	-0.16794	H	1
H72	0.02966	0.60731	0.2951	H	1
H73	1.00708	0.5609	0.23803	H	1
H74	0.57811	0.09318	-0.21968	H	1
H75	0.51781	0.10566	-0.03919	H	1
H76	0.61843	0.18506	-0.18605	H	1
H77	0.69262	0.38087	-0.15611	H	1
H78	0.72499	0.32825	-0.01087	H	1
H79	0.77167	0.34944	-0.01402	H	1

H80	0.77539	0.41464	-0.289	H	1
H81	0.72907	0.3931	-0.29997	H	1
H82	0.38083	0.69314	-0.15324	H	1
H83	0.33688	0.60739	-0.29663	H	1
H84	0.36248	0.58635	-0.28312	H	1
H85	0.42303	0.6526	-0.00959	H	1
H86	0.39694	0.6726	-0.00598	H	1
H87	0.13127	0.61185	-0.10643	H	1
H88	0.13278	0.60556	-0.34629	H	1
H89	0.1592	0.63279	-0.23879	H	1
H90	0.14836	0.48158	0.0235	H	1
H91	0.12613	0.48184	-0.13927	H	1
H92	0.12007	0.48131	0.10438	H	1
H93	0.61763	0.11229	-0.34155	H	1
H94	0.62352	0.11834	-0.09818	H	1
H95	0.64701	0.13911	-0.262	H	1
H96	0.51296	0.13641	0.10899	H	1
H97	0.50703	0.13775	-0.13185	H	1
H98	0.51611	0.16524	0.01161	H	1
O99	0.35676	0.42591	1.09848	O	1
H100	0.35137	0.41405	1.20787	H	1

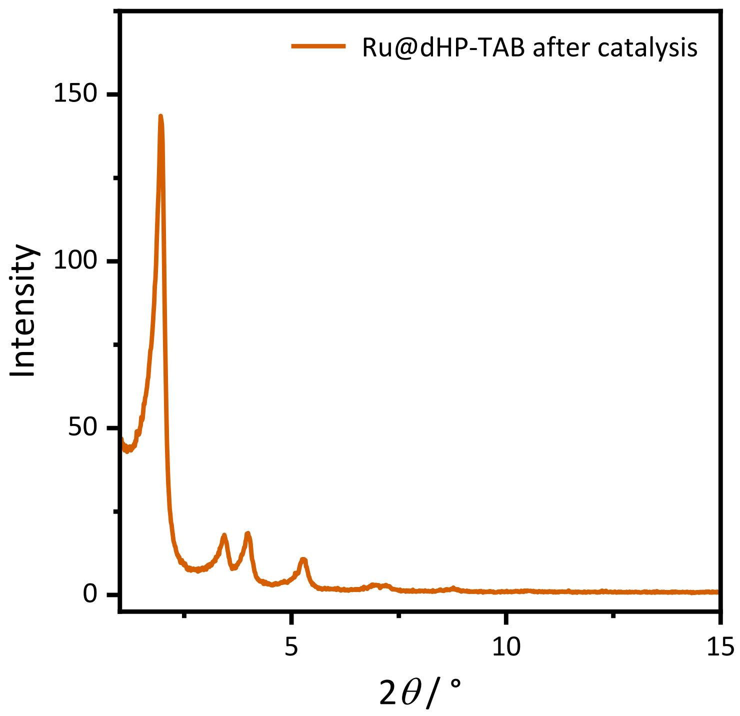

Figure S11. XRPD (λ = 1.789 Å) of **Ru@dHP-TAB** after catalysis.

S5 Gas Sorption Experiments

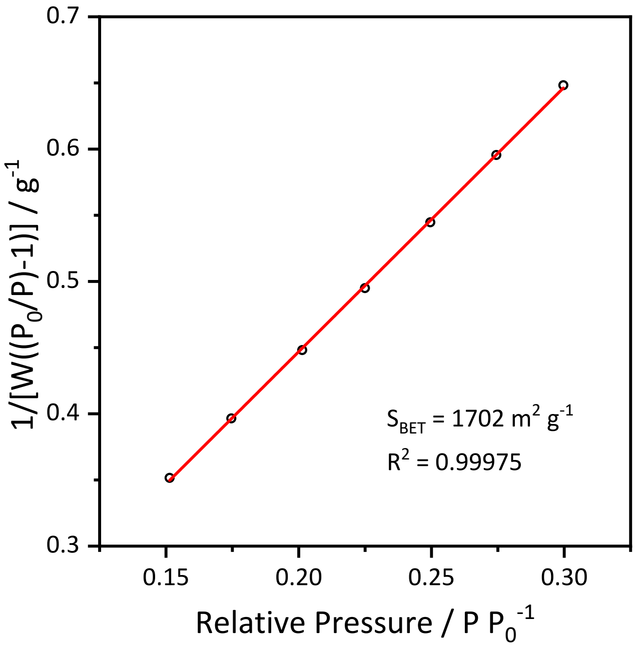

Figure S12. Multi-point BET surface area fit of **dHP-TAB COF** derived from N_2 sorption isotherm.

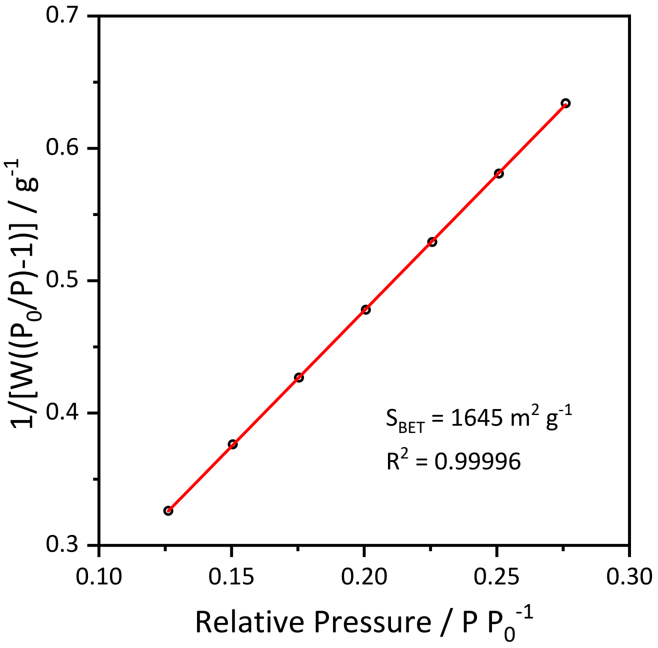

Figure S13. Multi-point BET surface area fit of **Ru@dHP-TAB COF** derived from N_2 sorption isotherm.

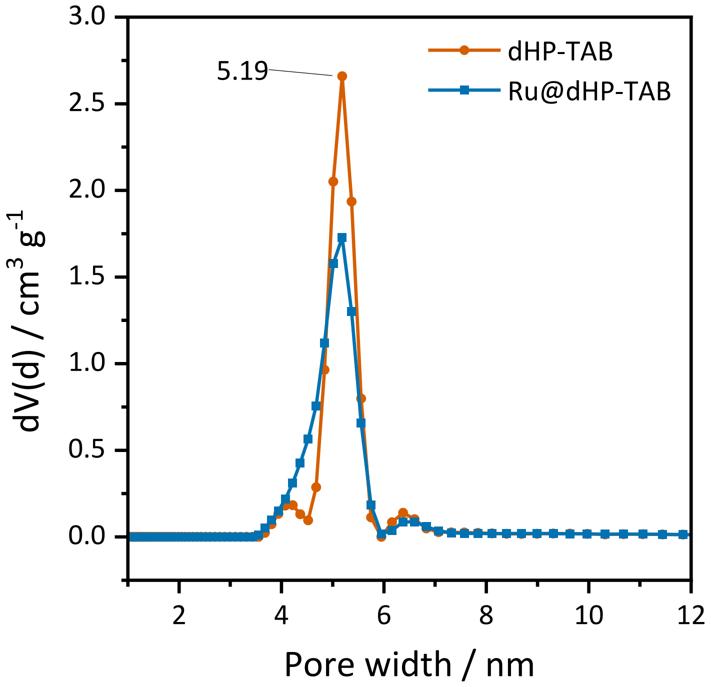

Figure S14. Pore size distribution obtained from the desorption branch of **dHP-TAB** and **Ru@dHP-TAB** after immobilization of the catalyst.

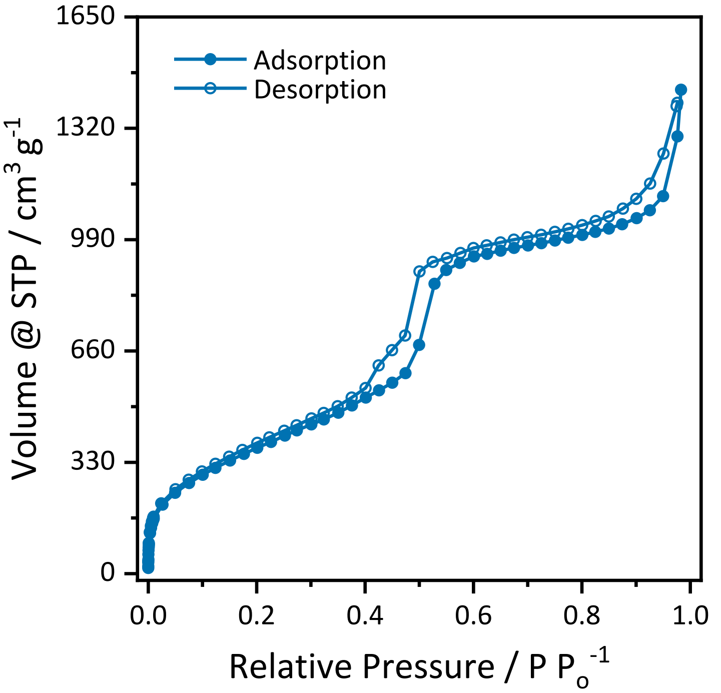

Figure S15. Nitrogen sorption isotherm at 77 K of **Ru@dHP-TAB** after catalysis.

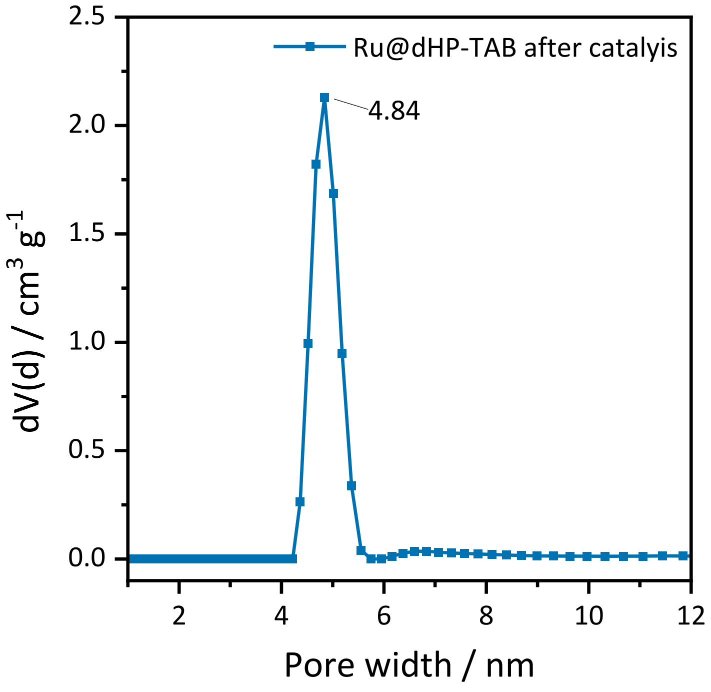

Figure S16. Pore size distribution obtained from the adsorption branch of **Ru@dHP-TAB** after catalysis.

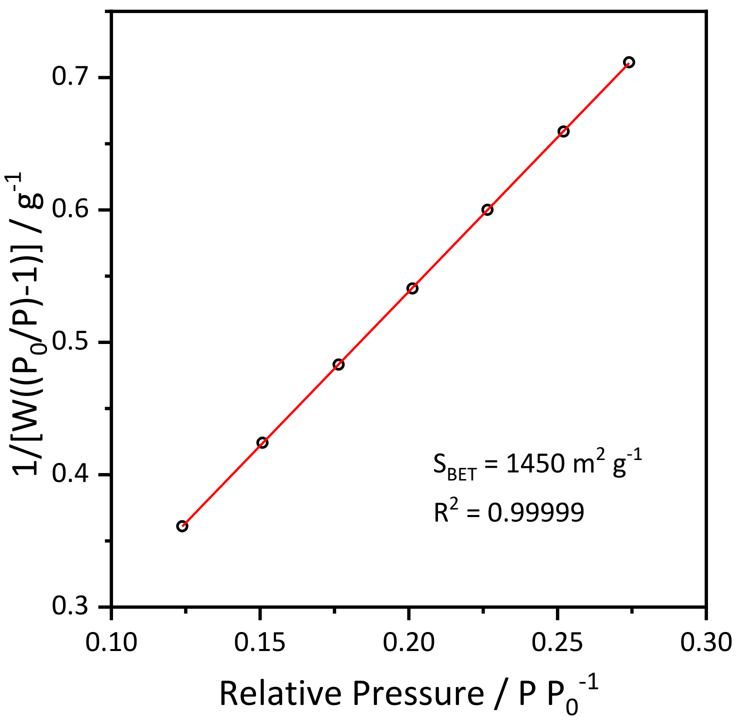

Figure S17. Multi-point BET surface area fit of **Ru@dHP-TAB COF** after catalysis derived from N_2 sorption isotherm.

S6 Solid State NMR

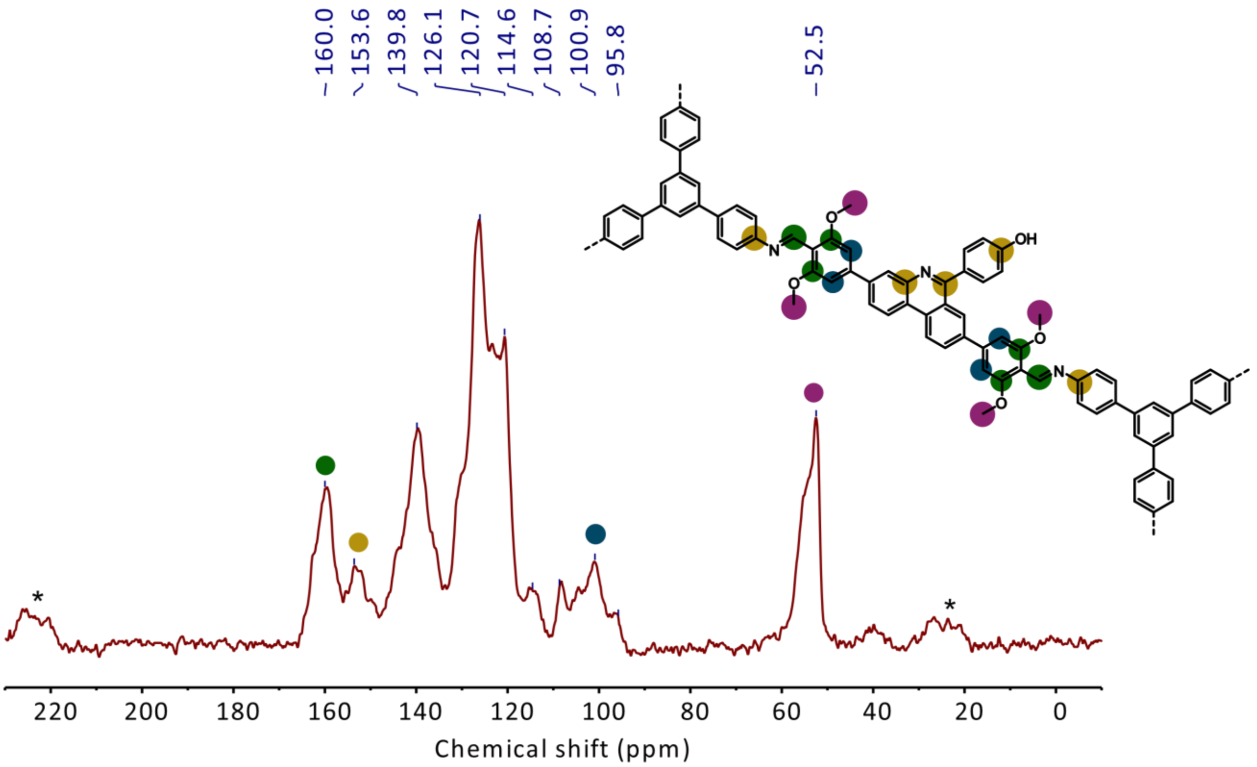

Figure S18. ^{13}C MAS ssNMR of **dHP-TAB COF**.

S7 XAS

Experimental details

All XAS experiments were carried out at PETRA III beamline P65 at Deutsches Elektronensynchrotron (DESY) in Hamburg, Germany. The measurements at the Ruthenium K-edge (22117 eV) were performed in transmission using a Si(111) double-crystal monochromator and a maximum synchrotron beam current of 100 mA. **Ru** in solid state (**Ru** solid) was studied as self-supporting wafer using boron nitride (BN) as binder. Spectra of **Ru** in solution (**Ru** solution) of benzene (50 mM) were recorded using a specially designed measurement cell allowing for protection gas atmosphere. The sample containing the immobilized complex on the COF (**Ru@dHP-TAB** solid) was measured without binder as powder sealed between Kapton foil. This sample was also measured as stirred suspension in benzene (**Ru@dHP-TAB** suspension). Sample preparation was carried out under inert atmosphere in a glove box. For energy calibration, a Ruthenium foil was used. Calibration was performed using the first inflection point in Ru K-edge XANES spectrum. The data acquisition was performed in continuous scan mode and rebinning with a window of 0.5 eV was applied. All measurements were carried out at room temperature.

Data analysis

In the first step of data analysis the background of the spectrum was removed by subtracting a Victoreen-type polynomial.[8–11] Due to the very differing shapes of the absorption edges of the samples and the used references, the first inflection point of the first derivative of the corresponding spectrum was defined as energy E_0. Afterwards a piecewise polynomial was used to determine the smooth part of the spectrum and was adjusted in a way that the low-R components of the resulting Fourier transform were minimal. The background-subtracted spectrum was divided by its smoothed part and the photon energy was converted to photoelectron wave number *k*. For evaluation of the EXAFS spectra the resulting functions were weighted with k^2 and calculated with *ARTEMIS* program. Curve fitting was performed using ab-initio-calculated phases and amplitudes from the FEFF8 program from the University of Washington. *ARTEMIS* works based on the EXAFS function and according to a formulation in terms of radial distribution functions:[8,12]

$$\chi(k) = \sum_j S_0^2(k) F_j(k) \int P_j(r_j) \frac{e^{\frac{-2r_j}{\lambda}}}{k r_j^2} \sin\left[2kr_j + \delta_j(k)\right] dr_j$$

The number of independent points N_{ind} was calculated according to information theory to determine the degree of overdeterminacy:[8]

$$N_{ind} = \frac{2\Delta k \Delta R}{\pi}$$

Here, *Δk* describes the range in *k*-space used for data analysis and *ΔR* corresponds to the distance range in the Fourier filtering process. The values used for the analysis are shown in Table S6. The quality of a fit was determined using two methods. The reduced χ^2_{red} considers the degree of overdeterminacy of the system and the number of fitted parameters *p*. It therefore allows a direct comparison of different models:[13]

$$\chi^2_{red} = \frac{({}^{N_{ind}}/_{N})}{N_{ind} - p} \sum_i \left(\frac{k_i^n}{\sum_j k_j^n \left| \chi_j^{exp}(k_j) \right|} \right)^2 (\chi^{exp}(k_i) - \chi^{theo}(k_i))^2$$

The R-factor, which represents the percental disagreement between experiment and adjusted function and takes into account both systematic and random errors according to the equation:[13]

$$R = \sum_i \frac{k_i^n}{\sum_j k_j^n \left|\chi_j^{exp}(k_j)\right|} \left|\chi^{exp}(k_i) - \chi^{theo}(k_i)\right| \cdot 100\%$$

The accuracy of the determined distances is 1 %, of the Debye-Waller-like factor 10 %[14] and of the coordination numbers depending of the distance 5-15 %. Initial values for coordination numbers and distances were adopted from Rietveld-analysis and afterwards iterated free in every fit as well as the Debye-Waller-like factor and the amplitude reducing factor.

XANES analysis details

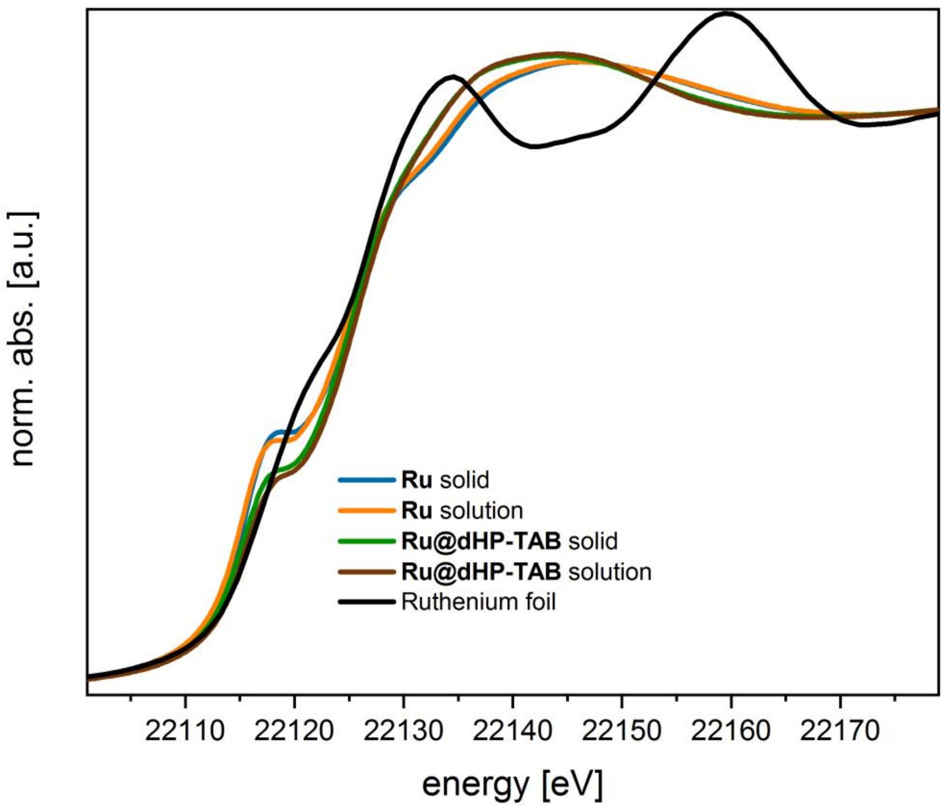

Figure S 19. XANES spectra of **Ru** in the solid state (**Ru** solid, blue), solution (**Ru** solution, orange), **Ru** immobilized in a mesoporous COF in the solid state (**Ru@dHP-TAB** solid, green), as suspension in benzene **Ru@dHP-TAB** solution, brown) as well as of the Ru(0) foil used for calibration (black).

The edge energy in a XANES spectrum gives information about the oxidation state of the analyzed complex. Metal centers with a higher oxidation number typically show a higher edge energy. The XANES spectra in Figure S14 reveal no edge energy shift compared to each other. This indicates that no changes in the oxidation state during the dissolution and immobilization of the homogeneous complex occur. The XANES spectra of Ru complexes (Figure S14) have the same edge energy of around 22115.4 eV. The absorption energy for the 1s electrons is in all cases around 1.6 eV lower than for the Ru(0)-foil (22117 eV, black line) that was used for the calibration of the spectra. Despite the lower edge energy, the XANES spectra of the Ru complex show nearly no edge shift compared to the Ru(0) foil, which is explained by ligand effects, since the formal ruthenium oxidation state in the complexes is +II. However, the two carbene ligands act as strong electron donors and increase the electronic density at the Ru center significantly, which could be responsible for the rather low absorption edge energy of the present compounds. In addition, the presence of an intense feature within the edge step overlaps with the edge step function, interfering with the edge step determination.

Table S5. Edge energies of the four Ru samples.

Sample	Edge Energy E_0 [eV]

**Ru** solid	22115.5
**Ru** solution	22115.4
**Ru@dHP-TAB** solid	22115.4
**Ru@dHP-TAB** solution	22115.8
Ru(0) foil	22117.0

EXAFS analysis details:

The k- and R-ranges applied in the analysis of the Ru complexes together with the corresponding fit parameters are summarized in Table S6. Figure S15 shows the Fourier-transformed EXAFS spectra of the four Ru complexes together with corresponding first shell scattering paths. Figure S16 illustrates the fitted function, experimental data, residual plot as well as first shell contributions for the Ru samples in k-space. The corresponding first shell scattering paths including coordination numbers, bond distances and Debye-Waller factors are collected in Table S7.

Table S6. k- and R ranges as well as corresponding fit parameters of the analysis of the four Ru samples.

sample	k-range [$Å^{-1}$]	R-range [Å]	R-factor	Reduced Chi-square	SO2 value
**Ru** solid	2.80 - 15.20	1.15 - 4.50	0.0025	59	1.0
**Ru** solution	2.60 - 14.70	1.15 - 4.50	0.0009	8	1.0
**Ru@dHP-TAB** solid	2.60 - 14.30	1.15 - 4.50	0.0047	7	1.0
**Ru@dHP-TAB** solution	2.78 - 14.70	1.15 - 4.50	0.0039	63	1.0

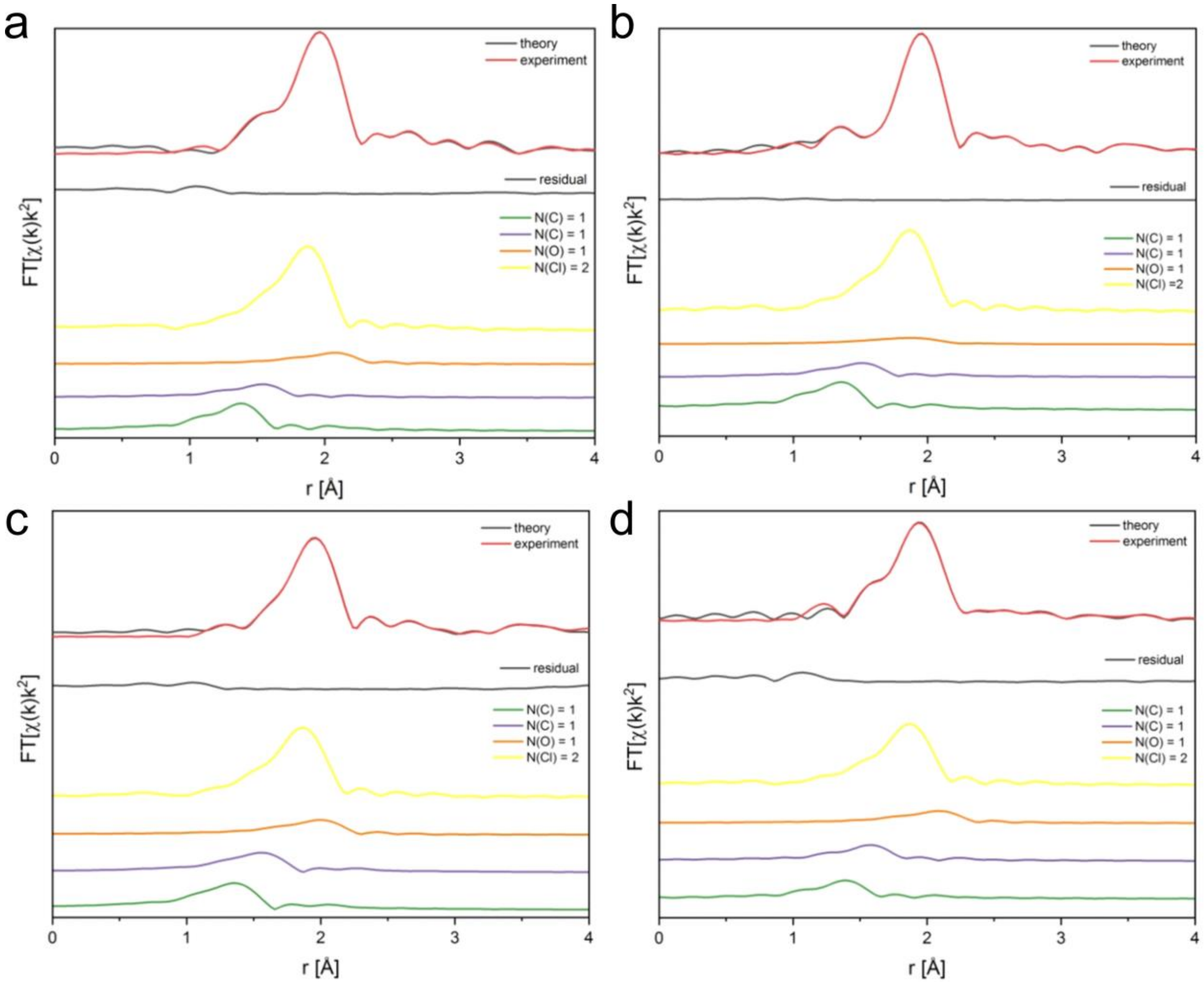

Figure S20. Fourier-transformed EXAFS spectra of the **Ru** in solid state and solution (benzene) as well as immobilized in a mesoporous COF. a) **Ru** solid, b) **Ru** solution, c) **Ru@dHP-TAB** solid, d) **Ru@dHP-TAB** solution.

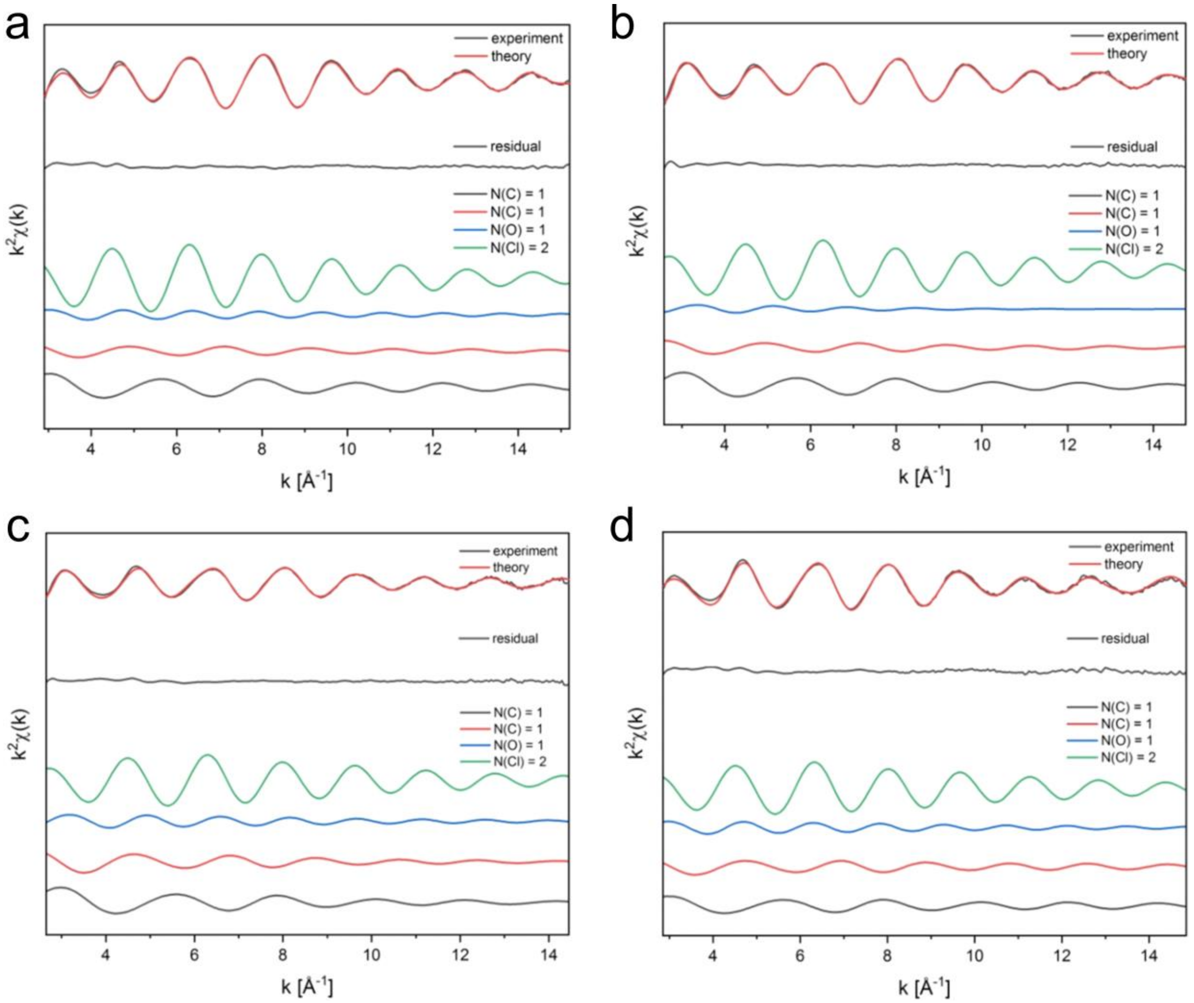

Figure S21. Fitted function compared with experimental data, residual plot and first coordination shell paths for the four Ru samples in k-space. a) **Ru** solid, b) **Ru** solution, c) **Ru@dHP-TAB** solid, d) **Ru@dHP-TAB** solution.

Table S7: First shell coordination Numbers (N), bond lengths (R + ΔR) and Debye-Waller factors of the measured samples.

Scattering paths	solid			solution/ suspension			Difference of bond lengths
	N	$R + \Delta R$ [Å]	σ^2 [Å^2]	N	N	$R + \Delta R$ [Å]	σ^2 [Å^2]
**Ru**				**Ru**			
Ru-C	1.0(1)	1.819(4)	0.0012(3)	Ru-C	1.0(1)	1.819(4)	0.0012(3)
Ru-C	0.7(1)	1.983(9)	0.0013(3)	Ru-C	0.7(1)	1.983(9)	0.0013(3)
Ru-O	0.8(1)	2.549(20)	0.0018(15)	Ru-O	0.8(1)	2.549(20)	0.0018(15)
Ru-Cl	2.4(0)	2.323(4)	0.0032(1)	Ru-Cl	2.4(0)	2.323(4)	0.0032(1)
**Ru@dHP-TAB**				**Ru@dHP-TAB**			
Ru-C	1.2(1)	1.836(6)	0.0036(3)	Ru-C	1.2(1)	1.836(6)	0.0036(3)
Ru-C	1.3(2)	2.051(11)	0.0040(4)	Ru-C	1.3(2)	2.051(11)	0.0040(4)
Ru-O	1.0(2)	2.491(17)	0.0028(9)	Ru-O	1.0(2)	2.491(17)	0.0028(9)
Ru-Cl	1.7(1)	2.326(4)	0.0031(2)	Ru-Cl	1.7(1)	2.326(4)	0.0031(2)

S8 Liquid State NMR

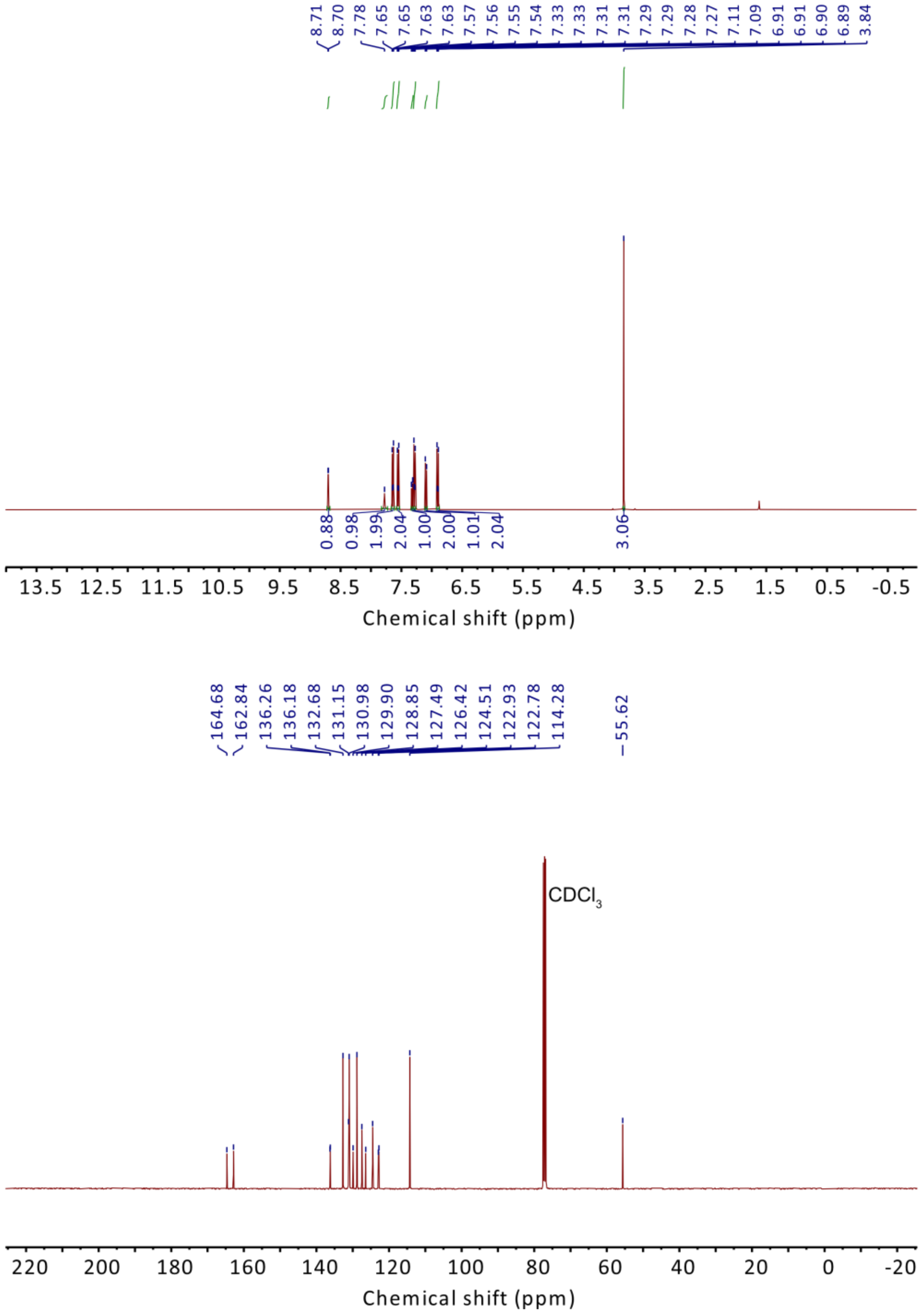

Figure S22. ^{1}H NMR (top) and ^{13}C NMR (bottom) of ***N*-(4,4'-dibromo-[1,1'-biphenyl]-2-yl)-4-methoxybenzamide (7)** in $CDCl_3$.

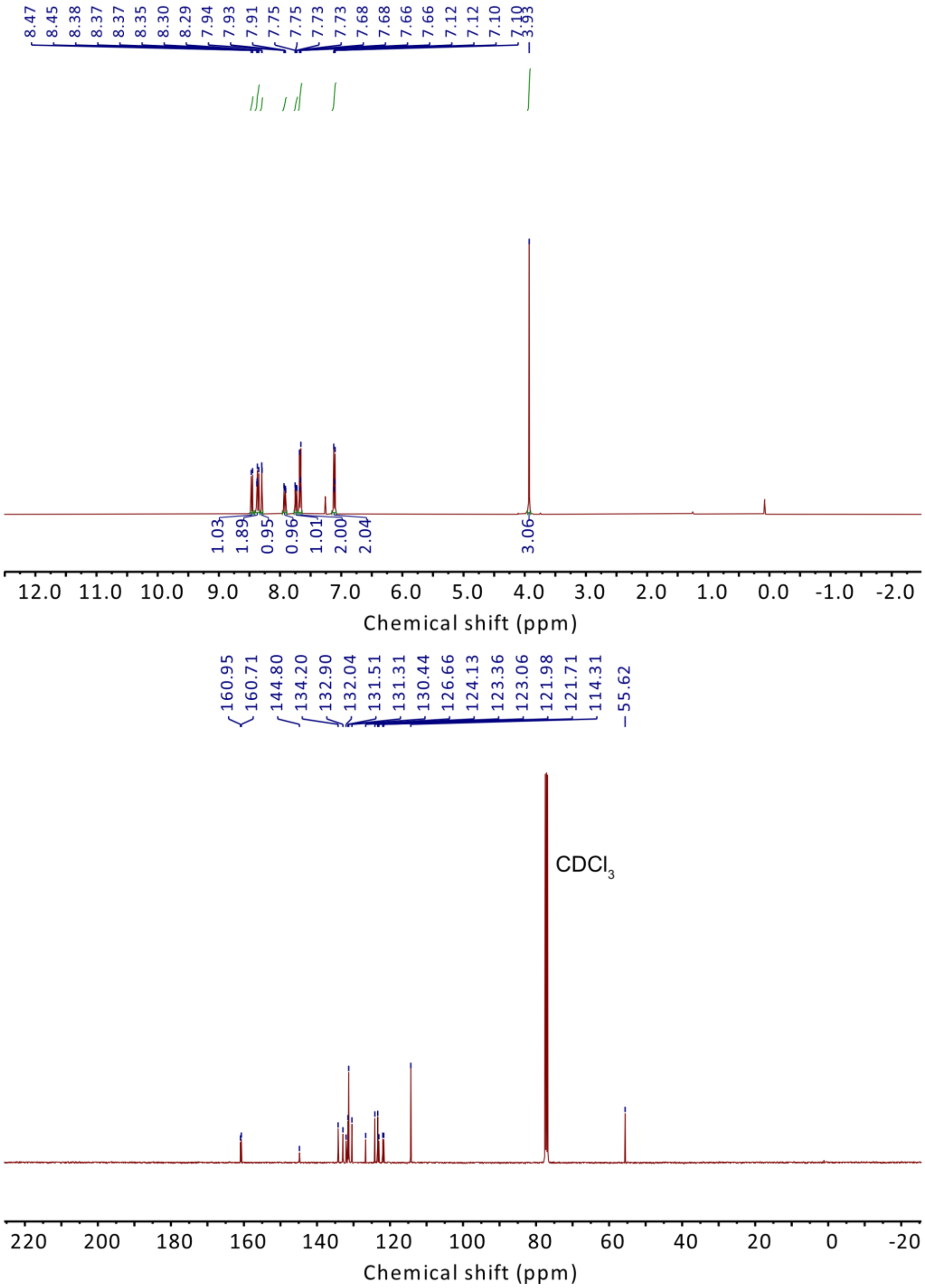

Figure S23. ^{1}H NMR (top) and ^{13}C NMR (bottom) of **3,8-dibromo-6-(4-methoxyphenyl)phenanthridine (8)** in $CDCl_3$.

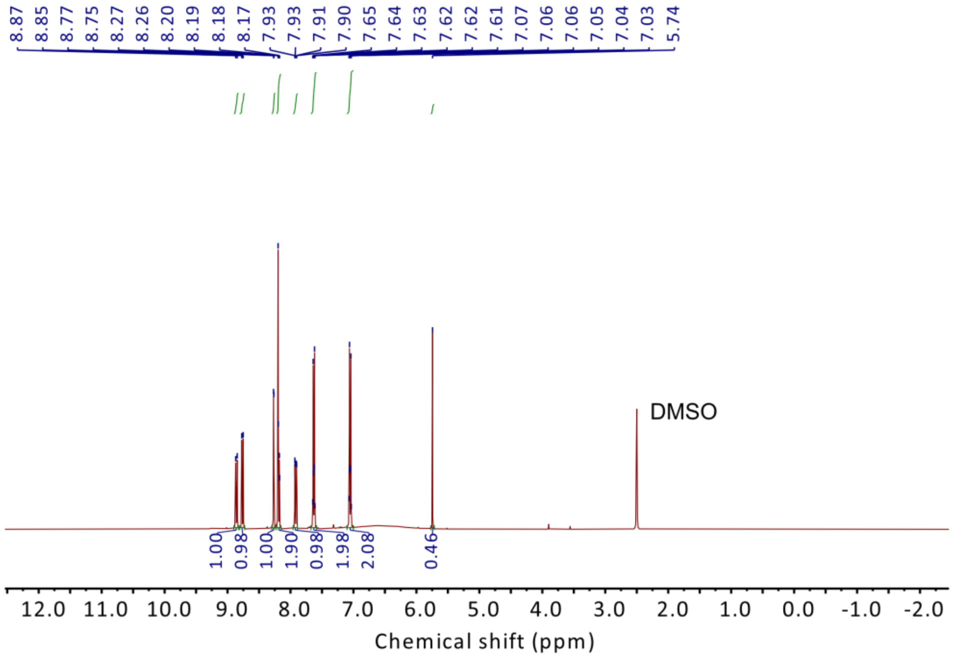

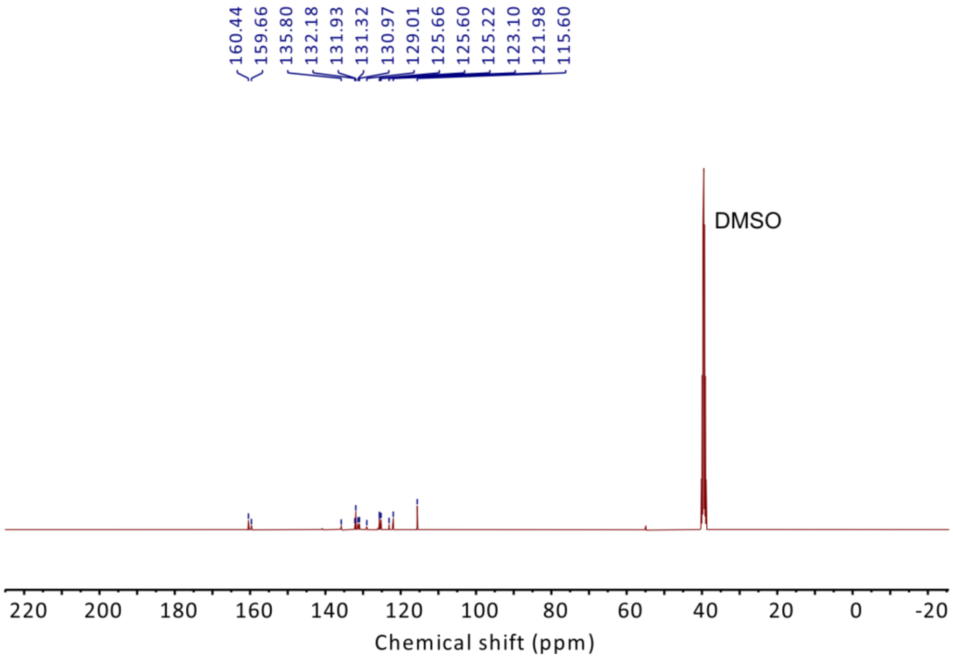

Figure S24. ^{1}H NMR (top) and ^{13}C NMR (bottom) of **4-(3,8-dibromophenanthridin-6-yl)phenol (9)** in DMSO.

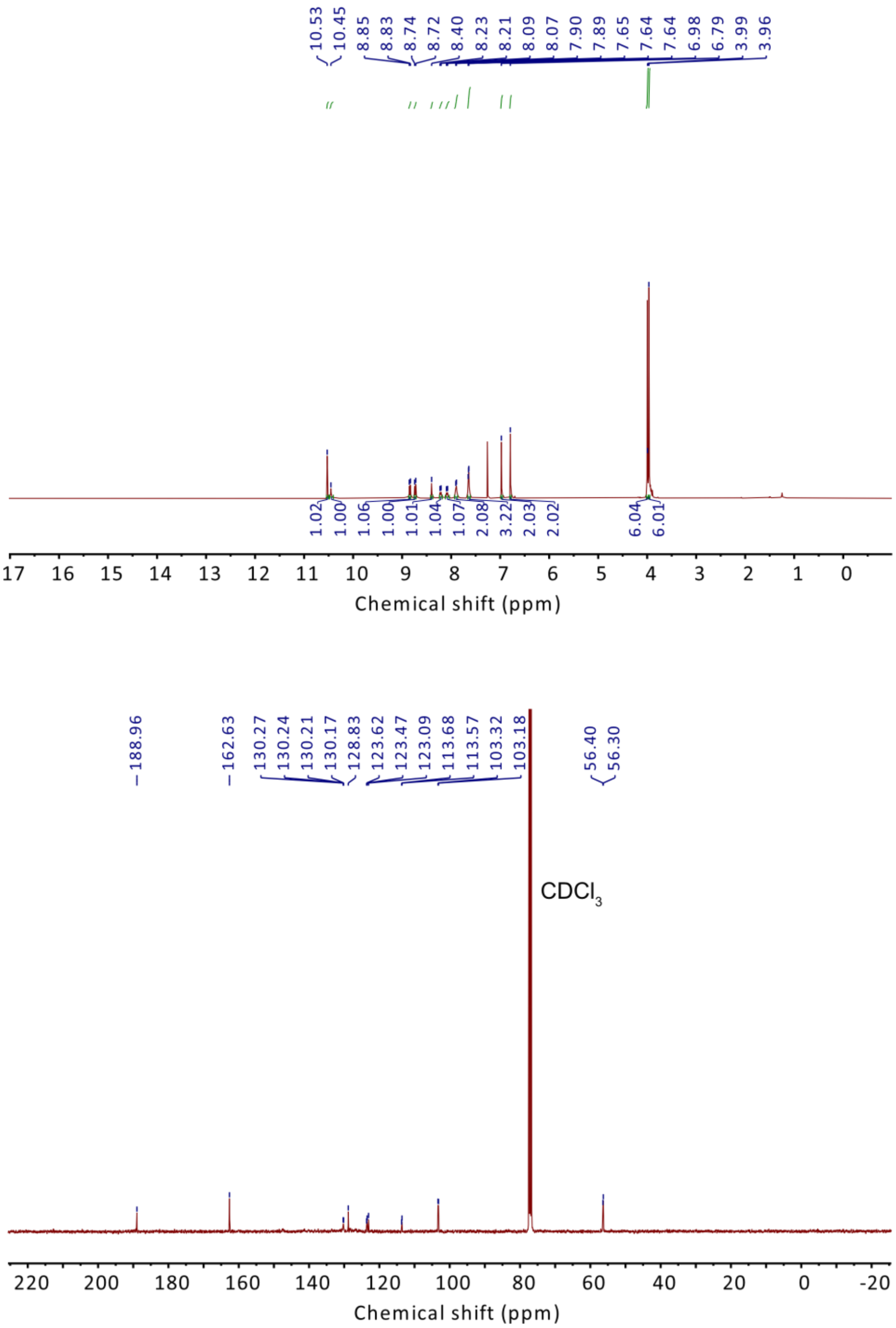

Figure S25. ^{1}H NMR (top) and ^{13}C NMR (bottom) of **4,4'-(6-phenylphenanthridine-3,8-diyl)bis(2,6-dimethoxybenzaldeyde) (dHP)** in $CDCl_3$.

S9 SEM/TEM and EDX analysis

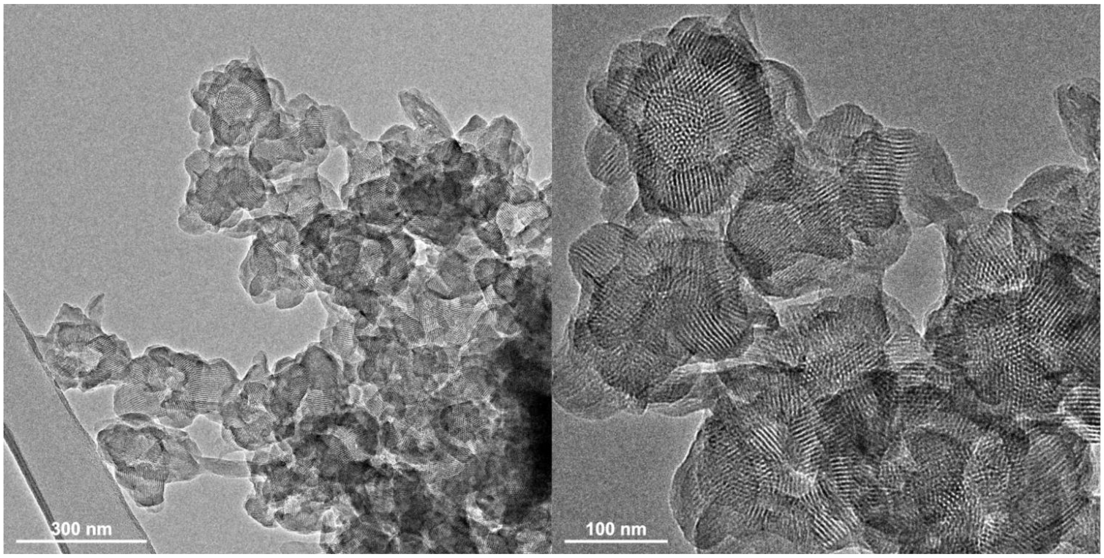

Figure S26. TEM images of **Ru@dHP-TAB COF**.

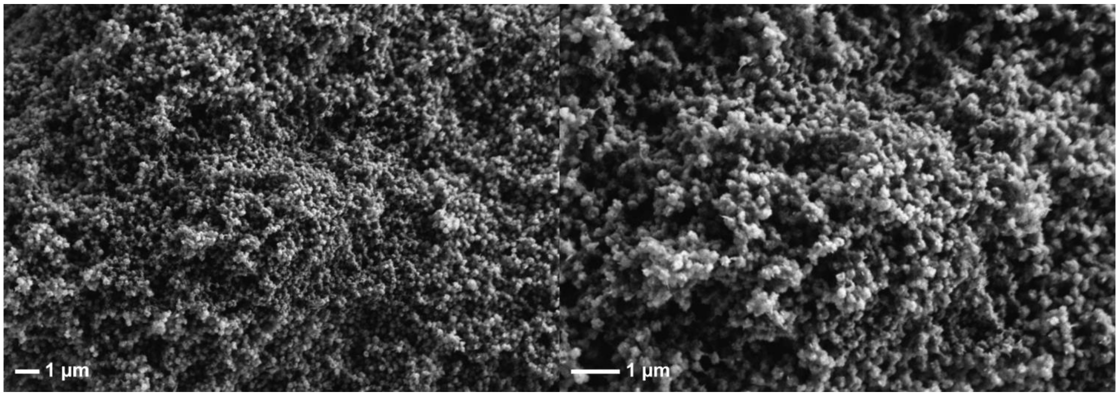

Figure S27. SEM images of **Ru@dHP-TAB COF**.

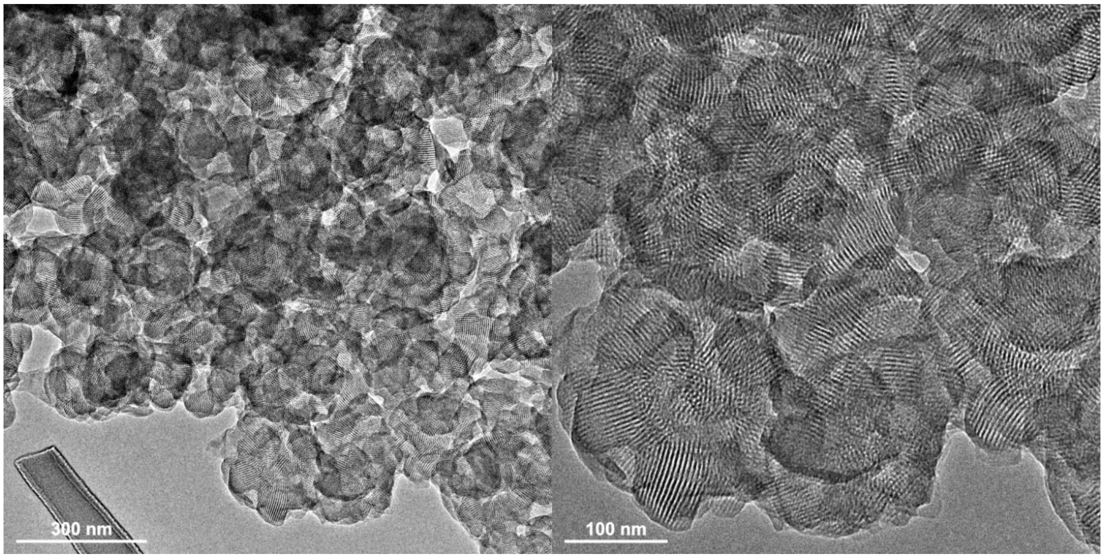

Figure S28. TEM images of **Ru@dHP-TAB COF** after catalysis.

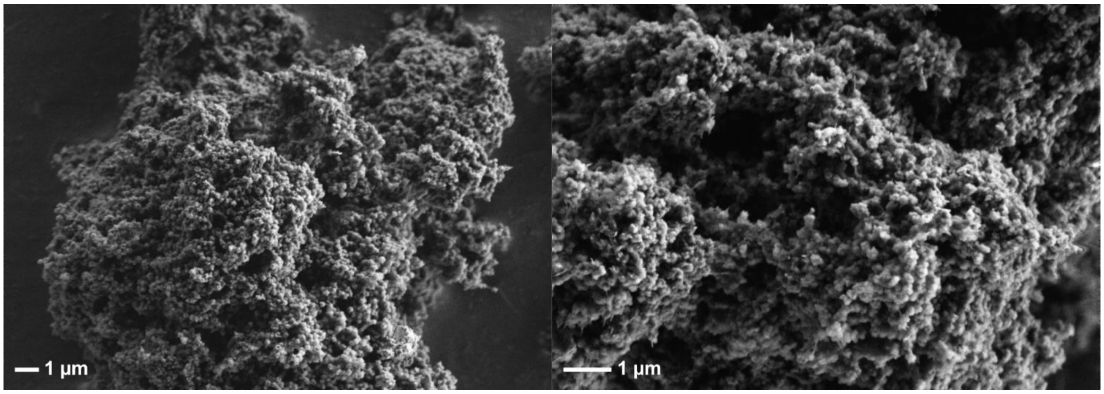

Figure S29. SEM images of **Ru@dHP-TAB COF** after catalysis.

S10 TGA analysis

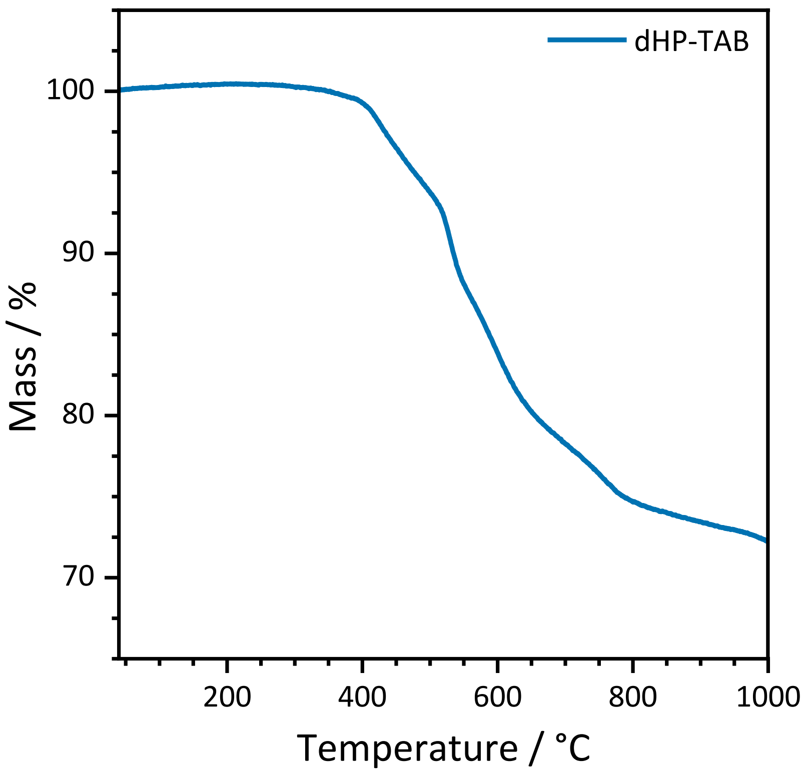

Figure S30. TGA measurement of **dHP-TAB** COF.

S11 References

(1) Emmerling, S. T.; Schuldt, R.; Bette, S.; Yao, L.; Dinnebier, R. E.; Kästner, J.; Lotsch, B. V. Interlayer Interactions as Design Tool for Large-Pore COFs. *J. Am. Chem. Soc.* **2021**, *0* (0).

(2) Fulmer, G. R.; Miller, A. J. M.; Sherden, N. H.; Gottlieb, H. E.; Nudelman, A.; Stoltz, B. M.; Bercaw, J. E.; Goldberg, K. I. NMR Chemical Shifts of Trace Impurities: Common Laboratory Solvents, Organics, and Gases in Deuterated Solvents Relevant to the Organometallic Chemist. *Organometallics* **2010**, *29* (9), 2176–2179.

(3) Chen, Y.; Li, F.; Bo, Z. Facile Synthesis of 3,8-Dibromo-Substituted Phenanthridine Derivatives and Their Conjugated Polymers. *Macromolecules* **2010**, *43* (3), 1349–1355.

(4) Movassaghi, M.; Hill, M. D. A Versatile Cyclodehydration Reaction for the Synthesis of Isoquinoline and -Carboline Derivatives. *Org. Lett.* **2008**, *10* (16), 3485–3488.

(5) Ziegler, F.; Teske, J.; Elser, I.; Dyballa, M.; Frey, W.; Kraus, H.; Hansen, N.; Rybka, J.; Tallarek, U.; Buchmeiser, M. R. Olefin Metathesis in Confined Geometries: A Biomimetic Approach toward Selective Macrocyclization. *J. Am. Chem. Soc.* **2019**, *141* (48), 19014–19022.

(6) Marx, V. M.; Herbert, M. B.; Keitz, B. K.; Grubbs, R. H. Stereoselective Access to Z and e Macrocycles by Ruthenium-Catalyzed Z-Selective Ring-Closing Metathesis and Ethenolysis. *J. Am. Chem. Soc.* **2013**, *135* (1), 94–97.

(7) Jee, J. E.; Cheong, J. L.; Lim, J.; Chen, C.; Hong, S. H.; Lee, S. S. Highly Selective Macrocycle Formations by Metathesis Catalysts Fixated in Nanopores. *J. Org. Chem.* **2013**, *78* (7), 3048–3056.

(8) Ravel, B.; Newville, M. ATHENA, ARTEMIS, HEPHAESTUS: Data Analysis for X-Ray Absorption Spectroscopy Using IFEFFIT. *J. Synchrotron Radiat.* **2005**, *12* (4), 537–541.

(9) Newville, M.; Liviņ, P.; Yacoby, Y.; Rehr, J. J.; Stern, E. A. Near-Edge x-Ray-Absorption Fine Structure of Pb: A Comparison of Theory and Experiment. *Phys. Rev. B* **1993**, *47* (21), 14126–14131.

(10) Newville, M. IFEFFIT: Interactive XAFS Analysis and FEFF Fitting. *J. Synchrotron Radiat.* **2001**, *8* (2), 322–324.

(11) Ertel, T. S.; Bertagnolli, H.; Hückmann, S.; Kolb, U.; Peter, D. XAFS Spectroscopy of Liquid and Amorphous Systems: Presentation and Verification of a Newly Developed Program Package. *Appl. Spectrosc.* **1992**, *46* (4), 690–698.

(12) Binsted, N.; Hasnain, S. S. State-of-the-Art Analysis of Whole X-Ray Absorption Spectra. *J. Synchrotron Radiat.* **1996**, *3* (4), 185–196.

(13) Bauer, M.; Bertagnolli, H. The Amplitude Reduction Factor and the Cumulant Expansion Method: Crucial Factors in the Structural Analysis of Alkoxide Precursors in Solution. *J. Phys. Chem. B* **2007**, *111* (49), 13756–13764.

(14) Koningsberger, D. C.; Mojet, B. L.; Van Dorssen, G. E.; Ramaker, D. E. XAFS Spectroscopy; Fundamental Principles and Data Analysis. *Top. Catal.* **2000**, *10* (3–4), 143–155.
